# Current Research of Graphene-Based Nanocomposites and Their Application for Supercapacitors

**DOI:** 10.3390/nano10102046

**Published:** 2020-10-16

**Authors:** Santosh K. Tiwari, Anukul K. Thakur, Amrita De Adhikari, Yanqiu Zhu, Nannan Wang

**Affiliations:** 1 Key Laboratory of New Processing Technology for Nonferrous Metals and Materials, Guangxi Institute Fullerene Technology (GIFT), Ministry of Education, School of Resources, Environment and Materials, Guangxi University, Nanning 530004, China; 2 Department of Printed Electronics Engineering, Sunchon National University, Chonnam 57922, Korea; anukulphyiitd@gmail.com; 3 Department of Chemistry, Ben-Gurion University of the Negev, Beer-Sheva 8410501, Israel; amrita.deadhikari.chem@gmail.com; 4 Department of Mathematics and Physical Sciences, College of Engineering, University of Exeter, London EX4 4QJ, UK

**Keywords:** graphene, supercapacitors, electrode materials, surface properties, electrolytes for supercapacitors

## Abstract

This review acmes the latest developments of composites of metal oxides/sulfide comprising of graphene and its analogues as electrode materials in the construction of the next generation of supercapacitors (SCs). SCs have become an indispensable device of energy-storage modes. A prompt increase in the number of scientific accomplishments in this field, including publications, patents, and device fabrication, has evidenced the immense attention they have attracted from scientific communities. These efforts have resulted in rapid advancements in the field of SCs, focusing on the development of electrode materials with features of high performance, economic viability, and robustness. It has been demonstrated that carbon-based electrode materials mixed with metal oxides and sulfoxides can perform extremely well in terms of energy density, durability, and exceptional cyclic stability. Herein, the state-of-the-art technologies relevant to the fabrication, characterization, and property assessment of graphene-based SCs are discussed in detail, especially for the composite forms when mixing with metal sulfide, metal oxides, metal foams, and nanohybrids. Effective synthetic methodologies for the nanocomposite fabrications via intercalation, coating, wrapping, and covalent interactions will be reviewed. We will first introduce some fundamental aspects of SCs, and briefly highlight the impact of graphene-based nanostructures on the basic principle of SCs, and then the recent progress in graphene-based electrodes, electrolytes, and all-solid-state SCs will be covered. The important surface properties of the metal oxides/sulfides electrode materials (nickel oxide, nickel sulfide, molybdenum oxide, ruthenium oxides, stannous oxide, nickel-cobalt sulfide manganese oxides, multiferroic materials like BaMnF, core-shell materials, etc.) will be described in each section as per requirement. Finally, we will show that composites of graphene-based electrodes are promising for the construction of the next generation of high performance, robust SCs that hold the prospects for practical applications.

## 1. Introduction

The intervention of energy in our daily life from the provision of hot water to the latest mobile gadgets is indispensable. Most of the energy supplied, to date, is harnessed from fossil fuels (such as natural gas, coal, and oil) [1]. However, the sources of fossil fuel are very limited, and their continuous use will result in global environmental changes by the release of a huge amount of CO_2_, CO, NO, SO_2_, etc., into the atmosphere [1,2]. Therefore, there is a “should” for us to switch to sustainable renewable energy sources (such as wind energy, solar energy, electrochemical energy, hydrogen energy, and geothermal energy) [3,4]. Renewable energy sources, such as sunlight, wind, and water, being constantly replenished naturally, can be utilized limitlessly without any significant side effects [1,5,6]. However, the application of these renewable energy sources is largely limited due to the lack of proper technology for storage and transport, [7,8] in addition to generation and conversion. According to the World Energy Council, the world needs to double its energy supply by 2050 to meet its demands, therefore substantial amounts of research projects have been diverted to the development and exploration of the various aspects of renewable sources of energy [2,3]. Among many distinguished renewable energy sources, electricity generated from the conversion of chemical energy is considered to be the most striking approach for modern applications [3,6,7,8]. In this aspect, the electrochemical energy production of electricity and its storage has been considered as an effective alternative power source to match the high electric energy demands of future scientific, sustainable, and eco-friendly modules [7,8]. Conventional capacitors, supercapacitors (SCs), Li-air batteries, and fuel cells are recognized as the most significant electrochemical energy-storage/-conversion systems [3,7,8]. In particular, the key features of SCs that are capable of handling high power rates compared with batteries and fuel cells have drawn enormous attention from both academia and industry [8,9]. Moreover to their excellent capacity of delivering high power, SCs are also notable for their long cycle life and swift charging-discharging rates, which in turn depends on how proficiently we consume electrode materials for the various kinds of SCs [10,11,12,13].

For electrochemical energy storage devices, advanced electrode materials play a pivotal role in defining their performance [14,15]. Hence, the design and fabrication of efficient electrode materials are of importance for the development of high-performance futuristic energy storage devices. Graphene-based materials with intriguing properties have shown to be a promising building block as electrode materials [16,17], and a wide range of graphene and its 2D analogues in conjunction with different additives, such as conducting polymers, metal oxides, core–shell structures, etc., could offer countless possibilities as electrodes for the construction of energy storage devices of improved performance [18]. It is because of the unique electronic conductivity of GO and its derivatives, which has been explored in detail elsewhere [19].

**Figure 1:**
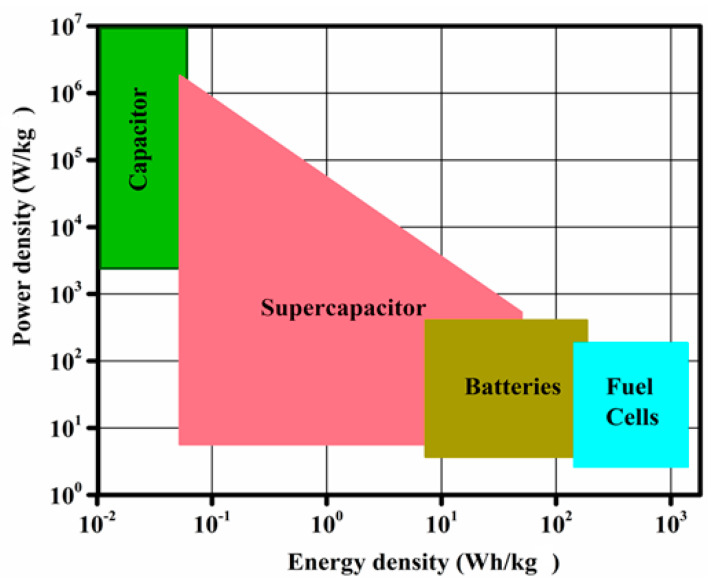
Ragone graph showing the performance of major energy-storage devices of batteries, capacitors, and electrochemical supercapacitors (SCs) in terms of their specific power and specific energy.

As shown in the schematic Ragone plot (Figure 1), the energy- and power-density values exhibited by SCs project them as a bridge between conventional capacitors and conventional batteries [8,10], offering both high power and energy densities. Figure 1 has also been utilized for the performance evaluation of different energy storage devices. In this graph, the values of specific energy are plotted versus specific power for the clear understanding of efficiencies of the fuel cell, batteries, SC, and capacitors [8]. Both axes of the graph are logarithmic, which permits the comparison of the performance of materials used in these different devices. Thus, the vertical axis of the Ragone plot defines how much specific energy is accessible, while the horizontal axis displays how speedily that energy can be delivered (i.e., power per unit mass can be obtained). For various comparative studies and discussion, the values provided in this review are mainly expressed in gravimetric scale, gravimetric capacitance (F/g), gravimetric energy density (Wh/kg), and gravimetric power density (W/kg), since gravimetric values are widely reported in the literature [20,21]. However, gravimetric values can sometimes be misleading in cases when they are determined at a very low mass loading (less than 1 mg of active mass per cm^2^), leading to false anticipation of the device performance, as explained by Simon and Gogotsi [10]. Thus, where needed, other scales were considered in this review for reality and lucidity.

Since the power density delivered by the SCs is very high compared with that of electrolytic batteries, while the specific energy delivered is much lower, different measures have been taken to enhance their energy density [7,8]. In the case of SCs, a wide range of materials can be used as electrodes, which have far more choices than the redox battery-electrode materials that are attributed to the charge storage realized through electrochemical kinetics via polarization resistance [7,8,10,11,12]. These SC devices can operate at a temperature as low as −40 °C, which is impossible for conventional batteries [10,11,12]. The energy-storage properties of various conventional popular devices are listed in Table 1, which highlights the superiority of SCs for various specific applications.

As discussed previously, several excellent reviews and books have been published to discuss the different aspects of SC’s materials. However, the rapid paces of recent development on SCs, especially on research associated with composites of a huge amount of combinations of new metal oxides/sulfide (nickel oxide, nickel sulfide, ruthenium oxides, stannous oxide, nickel-cobalt sulfide manganese oxides, multiferroic materials like BaMnF, other core–shell materials, etc.) with graphene and its analogues are exploding. These new forms of composites are considered as the most auspicious candidates for the next generation of high-performance SC materials. Therefore, in this review, we will focus specifically on these forms of graphene and its analogue-based nanocomposites consisting of oxides/sulfide, and provide a timely update about their performance as electrode materials in SCs. Firstly, we introduce some preliminary background information of SC to the beginners, which we hope will ease our review and briefly cover the results of previous literature published on graphene-based SCs, which mainly covered the working principle of SCs, the flexibility of electrodes, new technologies, nitrogen-doped graphene electrodes, conducting polymers, and inorganic hybrids with graphene materials for SCs [8,12,13,14,15,16,17,18]. Since the performances of an electrode material predominantly depends on factors like high surface area, electrical conductivity, and wetting of electrodes, whilst metal oxides with graphene and its derivatives have all characteristics above, therefore they possess great competence as electrode (high charging, high power density, discharging rate, high coulombic efficiency, and long cycling life) material for SCs. Thus, secondly, the surface properties of several new metal oxides/sulfide composites assembled with 2D nanostructures, which have not been analyzed previously, will be explored in-depth, for a better understanding of the mechanism behind the performance improvements achieved by these materials. Lastly, we sum up this article with an effort to highlight future directions of research, key challenges, and innovations that might occur for SC materials.

## 2. Working Principle of SCs: An Overview

**Figure 2:**
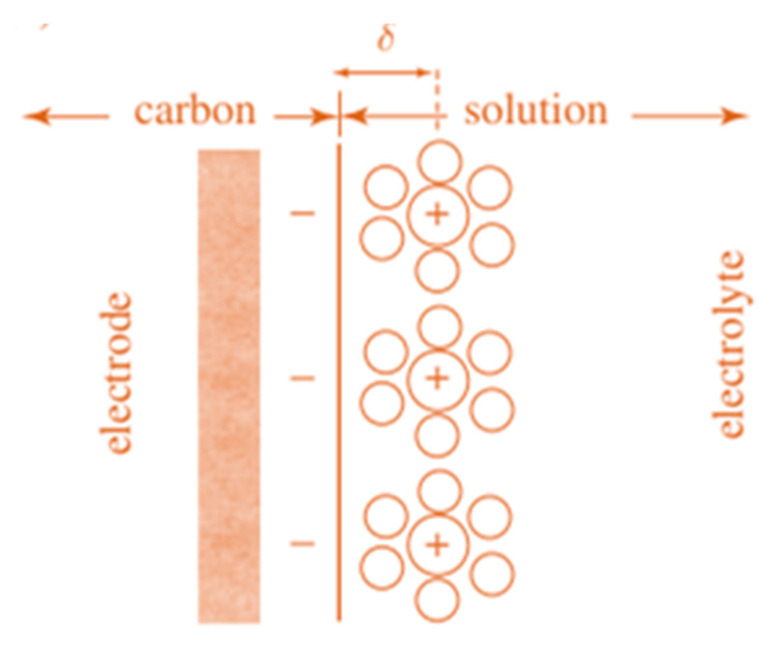
Cation adsorption onto the surface of a negatively polarized electrode, charging the double-layer capacitance (reproduced with permission from ref. [7]).

Each SC cell comprises of two electrodes, a separator and an electrolyte [13]. The electrodes can be similar, as in the case of symmetric cells; or different, as in the case of asymmetric cells. The separator is a thin ion-permeable material, which allows for the easy accessibility of ions, resulting in high ionic conductance. Apart from the separator, the conductivity of ions also strongly depends on the electrolyte used and can be further improved by carefully choosing a suitable electrolyte. SCs can store energy several orders of magnitude higher than that of the conventional capacitors (hence the name “super” or “ultra”) [6,14]. Appropriate cell design and intelligent selection of electrode materials for SCs are fundamental in the requisition of both high specific energy storage and power delivery, which would allow for SCs to act as stand-alone energy supplies for various applications [5,6,14]. Based on the mechanism of energy storage, SCs can be divided into two main categories, named I: redox SCs (or pseudo-capacitors) and II: electrical double-layer capacitors (EDLCs). This double-layer capacitance was first introduced by Helmholtz (1879) and adsorption of cations onto the surface of a negatively polarized electrode responsible for charging the double-layer capacitance as shown in Figure 2.

In general, capacitance can be formulated as

$$C=\frac{\in A}{d}$$

where, ∈ is the electrolyte dielectric constant, A is the surface area that the ions can access and d is the distance between the ions and the electrode surface.

It is very difficult to provide detailed illustrations on each effective material studied in the past five years for applications in high-performance SCs; however, we will try to summarize the present the key achievements of recent research associated with SCs. The discussion comprises of four main sections, which includes a mechanism, new materials, new devices, and future prospective. The first part presents the fundamental idea of SC, the second part is about recently developed electrode materials, including MOF (metal–organic framework), COF (covalent organic framework), black phosphorous, MXene (a group of 2D inorganic materials consists of a few atoms thick layers of transition metal carbides, nitrides, carbonitrides, etc.), metal nitrides, LaMnO_3_, M_x_MnO_2_, and RbAg_4_I_5_/graphite. The third part represents the area of device innovations, which includes different kinds of supercapacitors, referring to AC line-filtering supercapacitors, self-healing supercapacitors, shape-memory supercapacitors, thermal self-charging supercapacitors, etc. Finally, the last portion depicts the future prospects of these new materials and SC devices.

SCs belong to the first category, i.e., based on the redox reaction, which store charges both on the surface and in the bulk of the electrode material through highly reversible redox reactions, and are also termed as pseudocapacitors. In contrast, EDLCs store charges only on the surface of the electrode material non-faradaically, and the interaction between the charged electrode and electrolytic ions leads to the formation of an oppositely charged bilayer, and hence they are termed so [6]. The most commonly studied pseudocapacitive materials are transition-metal oxides (such as MnO_2_, V_2_O_5_, Co_3_O_4_ and SnO_2_) [11,12,13,15], polyoxometalates (MnCo_2_O_4_ and NiCo_2_O_4_) [16,17], metallocene (MCp_2_, M=Fe, Co, Mn, V and Cr) [17,18] and conducting polymers (polyaniline (PANI), polypyrrole (PPY), etc.) [18,19]. Unlike pseudocapacitance, EDLC is generally contributed by the carbonaceous materials with good conductivity and large surface areas, e.g., activated carbon, carbon nanotubes (CNTs), graphene, carbon black, etc. [7,8,10,11,12,15]. Thus, the key factors that determine the performance of a capacitor are the electrode materials and electrolytes [7,8]. Moreover, three vital parameters are significant for evaluating the performance of a SC device: capacitance—C_s_, operating voltage—V, and the equivalent series resistance (ESR).

## 3. Role of Graphene-Based Nanostructures for SCs

Graphene and its derivative are the most promising candidates for improving the performance of SCs, by enhancing the conduction properties of both electrodes and electrolytes [20,21]. Being proven as the most conductive material in the world [22], graphene nanosheets have been tested in high-performance electrodes, either as a monolith or as a valuable filler of a nanocomposite [23,24,25,26], whilst three-dimensional (3D) structures based on them have received special attention [27,28]. By forming a short conduction pathway in the electrode, the 3D structures have shown a direct impact on the conduction characteristics of the electrode [29,30].

Considering the high electrical conductivity, large specific surface area, and excellent mechanical properties of graphene, SCs prepared from graphene-based nanostructures are expected to exhibit very high performance and low final costs in the future [22,31]. In theory, graphene-based SCs are expected to have an upper capacitance limit of 550 F/g (with a theoretical specific surface area of 2630 m^2^/g for single-layer graphene nanosheets) [32]. When designed properly, nanostructures based on graphene can show excellent flexibility, which is an important feature for future flexible gadgets and energy-storage devices [33,34]. Owing to the fast ion transportation, graphene-based SCs can exhibit high power, long stability, and high-energy-density features [35], by using nonporous graphene electrodes [36,37]. Final properties of graphene-based porous nanostructures, including electrical, mechanical, and microstructural properties, can be easily engineered through the fabrication process [27]. Low-cost graphene-based nanostructures can be made by the hydrothermal reduction of graphene oxide (rGO) nanosheets, and they have been reported to introduce flexibility into all-solid-state SCs [38].

As discussed, different graphene derivatives have been assessed in the fabrication of high-performance SCs. Doping graphene with nitrogen results in an enhancement in the free charge-carrier density and the accessibility of the nanosheets surface area to the electrolyte solution [39]. Consequently, SCs with enhanced characteristics can be fabricated by employing nitrogen-doped (N-doped) graphene nanosheets and graphene porous nanostructures [40,41,42]. Moreover, due to the larger size of sulfur than that of carbon, the polarization of electron pairs via codoping of graphene with S and N enhances both the electrochemical and electrical properties of graphene-based SCs [43].

In addition to the aforementioned N- and S-doped nanosheets, functionalized-rGO and -graphene nanosheets and aerogels have also been documented for the fabrication of high-performance SCs [44,45,46,47,48]. Up to now, a number of review articles have been published on traditional SCs, especially on different electrode materials and on various kinds of electrolytes for SCs [49,50,51]. Through this review, however, our focus will be just on graphene-based electrodes and electrolytes for SCs.

## 4. Recent Trends in the Study of Electrolytes for SCs

An electrolyte is a chemical substance that produces an electrically conducting solution when liquefied in a suitable solvent such as water or ammonia [49]. The liquefied electrolyte splits into cations and anions, which disperse homogeneously over the solvent. Electrically, these electrolytic solutions are neutral. Therefore, if an electric potential is applied to the electrolytic solution, the cations of the solution will be attracted to the electrode that has an abundance of electrons, while the anions will be attracted to the electrode that has a deficit of electrons [49,50,51]. Such opposite anion and cation movements within the solution define the quantities of an electric current [49]. This phenomenon is possible with most soluble salts, acids, and bases [49,50,51]. However, some gases, such as hydrogen chloride, under suitable conditions (high temperature/low pressure) can also act as electrolytes.

In the case of batteries, SCs, etc., electrode materials are placed in an electrolyte, and when the desired voltage is applied the electrolyte will conduct electricity. Only electrons generally cannot pass through the electrolyte; instead, a chemical reaction occurs at the electrode (cathode), providing electrons to the electrolyte itself. Consequently, an additional reaction occurs at the anode, consuming electrons from the electrolyte [49]. Thus, due to these reactions, a negative charge cloud develops in the electrolyte surrounding the cathode, and a positive charge develops around the anode. The ions in the electrolyte neutralize these charges, enabling the electrons to keep flowing and the reactions to continue [49,50].

This explains why electrolytes have been identified as one of the most important influential components for the performance of SCs [49]. There are certain requirements for electrolytes associated with specific applications in SCs, as follows: (i) expanded potential window, (ii) high electrochemical stability, (iii) wide operating temperature range, (iv) high ionic conductivity, (v) low volatility and inflammability, (vi) environmental-friendly, and (vii) low cost. The electrolytes play a significant role in the EDL formation in the EDLCs and the redox reactions occurring in the pseudocapacitors [49,50,51]. Factors influencing the nature of electrolytes are summarized as follows: (i) the ion type and size, (ii) the interaction between ions and solvent molecules, (iii) the concentration of ions and solvent, (iv) the interaction between the electrolyte and electrode materials, and (v) the potential window that the electrolyte can tolerate [49,50,51]. The electrolytes can be broadly classified into liquid electrolytes and solid/quasi-solid-state electrolytes [50].

**Figure 3:**
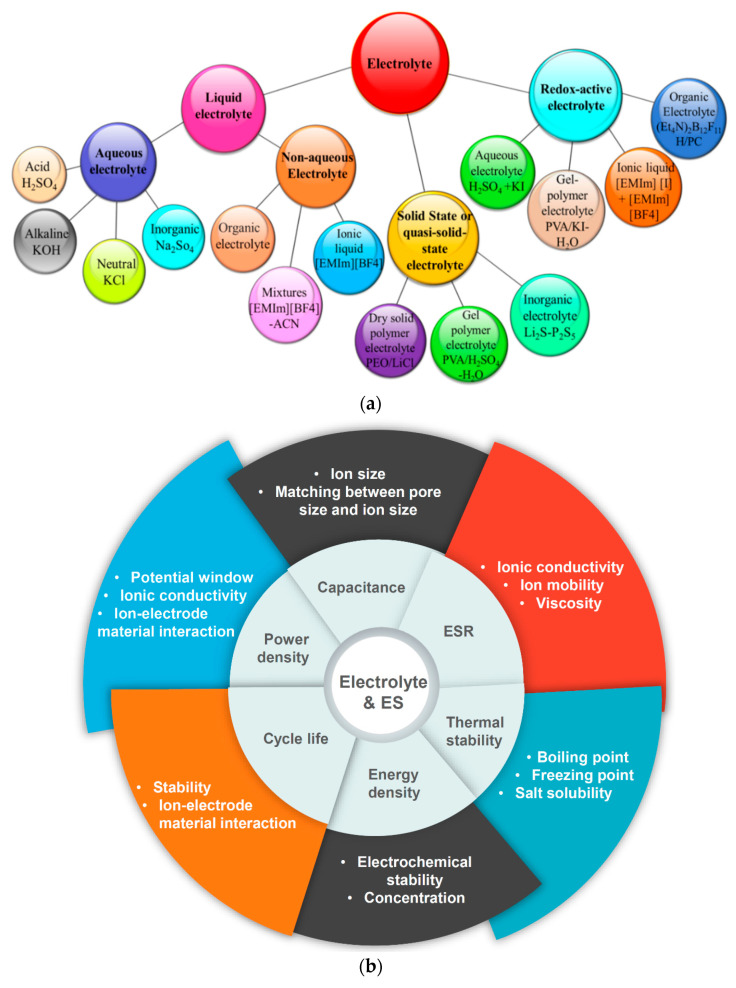
(**a**) Different types of electrolytes suitable for specific SC applications and (**b**) impact of the electrolyte on the performance of electrochemical SCs.

Liquid electrolytes can be divided into aqueous electrolytes, organic electrolytes, and ionic liquids (ILs); and solid-state electrolytes can be classified as organic electrolytes and inorganic electrolytes. However, to date, no electrolyte is faultless and meets all of the requirements, as each electrolyte has its own advantages and disadvantages. Figure 3a shows various types of electrolytes used in SCs. In other words, we can say that each class of electrode materials for SCs requires a specific class of electrolytic solutions. The key impacts of the electrolytes on the working performance of SCs are presented in diagrammatic form as Figure 3b, for the sake of simplicity and further reading [49].

### 4.1. Aqueous Electrolytes

Aqueous electrolytes have many advantages over non-aqueous solvents with respect to their electrochemical behavior. The aqueous electrolytes provide higher power densities than those of organic electrolytes. The protons in the aqueous electrolyte exhibit the highest mobility and are small in size and can be adsorbed to a single oxide ion [52]. The aqueous electrolytes may be acidic, alkaline, or neutral [53]. The working potential window of acidic and basic electrolytes is generally less than 1 V compared with neutral electrolytes, which is typically approximately 1.6–2.2 V [54]. The specific capacitance is obtained as the combined contribution of cations and anions presented in the electrolyte. They generally exhibit conductivity higher than that of organic or ionic liquids. Table 2 summarizes various aqueous-electrolyte-based SCs and their electrochemical performance [52,53]. However, a typical disadvantage of the aqueous electrolytes is their narrow potential windows, which originates from water that is generally electrolyzed at 1.23 V [54]. For this reason, there is a need to develop other electrolytes based on organic or ionic liquids towards wide potential windows [52,53].

### 4.2. Organic Electrolytes

For modern SC technologies, organic electrolytes are an imperative and key factor in the fabrication of batteries, capacitors, and SCs. These electrolytes are typically comprised of inorganic soluble chemicals dissolved in an organic solvent of very low electrical conductivity. Although at present most studies are directed towards the aqueous-electrolyte-based SCs in laboratory-scale innovation, the organic-electrolyte-based SCs are dominating the industrial-scale SC fabrication, and the commercial market prefers to use their high operation potential window capabilities, typically in the range of 2.5–2.8 V [49]. It is reported that increased operation cell voltages could deliver a momentous improvement in both energy and power densities. Furthermore, exhausting organic electrolytes permit the use of cheaper materials (a number of organic molecules) for the current collectors and packages. The electrolytes for commercial EDLCs consist of some conductive salts, such as tetraethylammonium tetrafluoroborate (TEABF4), which is dissolved in acetonitrile (ACN) and polycarbonate (PC). Table 3 lists several organic electrolytes used in different SC devices. However, the notable disadvantages of organic electrolytes are their high cost, low conductivity, smaller specific capacitance, volatility and toxicity, and self-discharge behavior.

### 4.3. Ionic Liquids

Ionic liquids (any salt materials that melt without vaporizing or decomposing typically yields an ionic liquid) are a very important class of materials and have attracted significant research interests worldwide [55]. Ionic liquids have various uniqueness and are becoming increasingly more attractive for emerging applications. They are composed of ions (cations and anions) with melting points below 100 °C. Table 4 summarizes list of ionic liquid used in previous studies. Ionic liquids play a very crucial role in the construction of different SCs, including hybrid SCs and batteries, as an electrolyte [53,54,55]. Herein, the basic criteria regarding the prototypical applications of ionic electrolytes specifically for SCs will be discussed, and the advantages and disadvantage summarized.

Furthermore, ionic liquids possess the following key advantages [53,54,55]: high thermal and electrochemical stability, non-inflammability, negligible volatility, a high operative voltage (above 3 V) even greater than that of organic electrolytes, and tunable physical and chemical properties.

Ionic liquids are classified as three major types: (1) protic (ionic liquid that comprises of a labile H^+^), (2) aprotic (ionic liquid molecules do not have a hydrogen atom attached to an atom of an electronegative element), and (3) zwitterionic (molecules consisting of two or more functional groups in ionic form) [55]. They are all good candidates for SC applications, especially for those working on double-layer charging. One of the most important advantages of ionic liquids is that they can be considered as solvents, and thus, their liquidity is very important. Prior to realizing grid-scale applications of energy-storage devices, several key issues remain, including the development of inexpensive, high-performance materials and electrolytes, which are ecofriendly and compatible, even with low temperature and large-scale processing. The given data provide some of the limits of ionic liquids for electrochemical application. There are still certain disadvantages of ionic liquids, including the following: the choice of ionic liquids is not very simple compared with other electrolytes, the high viscosity of the liquids limits their use for commercial purposes, the cost of purifying liquids is very high, handling requires much care, and they are mostly expensive and not ecofriendly.

Existing reports have shown that the specific capacitances for graphene-based materials in H_2_SO_4_ electrolyte are greater than those using neutral electrolytes [74,75]. Apart from the specific capacitance, the ESR of the resulting solid-state supercapacitors (SSSCs) in H_2_SO_4_ electrolyte is lower than in neutral electrolytes, which is attributed to the larger ionic conductivity of H_2_SO_4_. Furthermore, the specific capacitances for pristine graphene in the neutral electrolyte are also lower compared with those in the H_2_SO_4_ electrolyte [49]. Meanwhile, many reports have revealed similar specific capacitances and energy densities when using H_2_SO_4_ or aqueous KOH as inorganic electrolytes. It is noteworthy that graphene-based SCs in organic electrolytes exhibit lower specific capacitances than in inorganic electrolytes [76]. This reduced value of EDL capacitance can be attributed to the larger solvated ion sizes and small dielectric constants [49].

## 5. Advanced Electrodes for SCs: Present Status and Prospects

The detection of the opportunity for storing electric charges on the surface of materials arose from phenomena linked with rubbing of stone during prehistoric times [77,78]. However, the real scientific proof of surface charge accumulation was not properly understood until 1957. When a group of electrical engineers was investigating devices using porous carbon electrode, they realized the electric double-layer capacitor effect [77]. To date, considerable research has focused on electrode materials for SC applications; however, continuous investigations and innovative approaches are required to enhance the SC performance that allows for supplying quick bursts of energy, which are in high demand for the next generation of electronic devices [77,78].

To date, several research frontiers in energy storage have led to the development of electrode materials with superior electrochemical performance. For example, Wu et al. have reported the synthesis of hydrous RuO_2_/graphene sheets with different ruthenium loadings, which exhibited a specific capacitance of 570 F/g [79,80]. Besides, Bi et al. increased the capacitive performance of RuO_2_ using RuO_2_/CNT nanocomposites and achieved a specific capacitance of 935 F/g [79]. However, the commercial application of RuO_2_ has been restricted due to its high cost. Hence, cheap metal oxides, such as MnO_2_, NiO, SnO_2_, etc., have been increasingly studied to obtain an ideal replacement for the expensive RuO_2_ [79,80,81,82,83,84]. We will summarize these efforts in detail in the following subsections.

### 5.1. Metal-Based Electrodes

Many studies have reported various metal oxides mixed with graphene or rGO composites for the construction of electrochemical electrodes. The Mn_3_O_4_/graphene nanocomposites synthesized by Wang et al. exhibited a specific capacitance of 175 F/g in 1 M Na_2_SO_4_ and 256 F/g in 6 M KOH [85]. He et al. synthesized CoFe_2_O_4_/rGO nanocomposite SC electrodes and obtained a specific capacitance of 123.3 F/g [86]. Nagaraju et al. reported V_2_O_5_/rGO nanosheet electrodes possessing an impressive specific capacitance of 635 F/g at a current density of 1 A/g [87]. Flower-like NiO/rGO synthesized by Li et al. exhibited a specific capacitance of 428 F/g at a current density of 0.38 A/g [83]. Zhang et al. reported the synthesis of CdS/rGO nanocomposites and documented a specific capacitance of 300 F/g [82,83]. Metallic cellulose paper-based electrodes have been reported by Ko et al. for SC applications, which exhibited a maximum power of 15.1 mW cm^−2^ [88].

Most of the measurements of the aforementioned systems were carried out in the conventional three-electrode configuration. However, a review by Stoller and Ruoff suggested that the three-electrode configuration is valuable for the determination of electrochemical-specific material characteristics, while a two-electrode configuration portrays the physical configuration, charge transfer, and internal voltage of packaged SCs, thus providing better information on the electrode materials [89]. Jiang et al. reported the in situ incorporation of GO flakes and PEDOT: PSS into the bacterial nanocellulose (BNC) matrix and their electrode materials exhibited a specific capacitance of 373 F/g at a current density of 1 A/g [90]. Layered double hydroxides (LDHs) have recently been intensely studied as a candidate for SC applications. Wang et al. studied the performance of cobalt nickel iron–LDH/carbon nanofibers and activated carbon in asymmetric SCs, and reported their excellent performance [91]. Peng et al. synthesized CoAl-LDH/fluorinated graphene composites and reported an even higher specific capacitance of 1222 F/g at 1 A/g, with a very good rate capability [90,91].

To date, various studies have been carried out by different research groups, which undoubtedly confirmed the huge potentials of these new composite electrode materials in a wide range of SCs suitable for future energy-storage devices, however huge data inconsistency remains to be pointed out. In a three-electrode setup, the tiny amount of composite attached to the electrode to be tested imposes large uncertainty on the final outcome. In this review, it is impossible to cover all recent progress, but we collected some important works and presented them in Table 5 to highlight the different electrode materials and their performance in SCs.

The utilization of carbonaceous materials as electroactive materials has multiple advantages, such as (i) high specific surface area, (ii) low cost, (iii) wide availability, and (iv) mature electrode production technologies. Having all these features, graphene has logically attracted huge research interest in SCs since its discovery. Graphene is a single-layer *sp*^2^-hybridized carbon layer with a honeycomb structure, exhibiting many unique properties, including high carrier mobility, high thermal conductivity, and strong mechanical behavior. However, the valence and conduction bands of graphene are overlapped, which hinders its direct use in electronic applications. To overcome this, it is essential to generate band gaps and tune the activity in graphene. Another challenge for graphene to be used in electronic applications lies in the π–π stacking interactions of the graphitic sheets, which tends to result in their self-aggregation [108]. To counter this aggregation challenge, the distribution of metal/metal oxide over the graphitic sheets could be a useful strategy, which could also increase the surface area and conductivity of the graphitic sheets [109].

**Figure 4:**
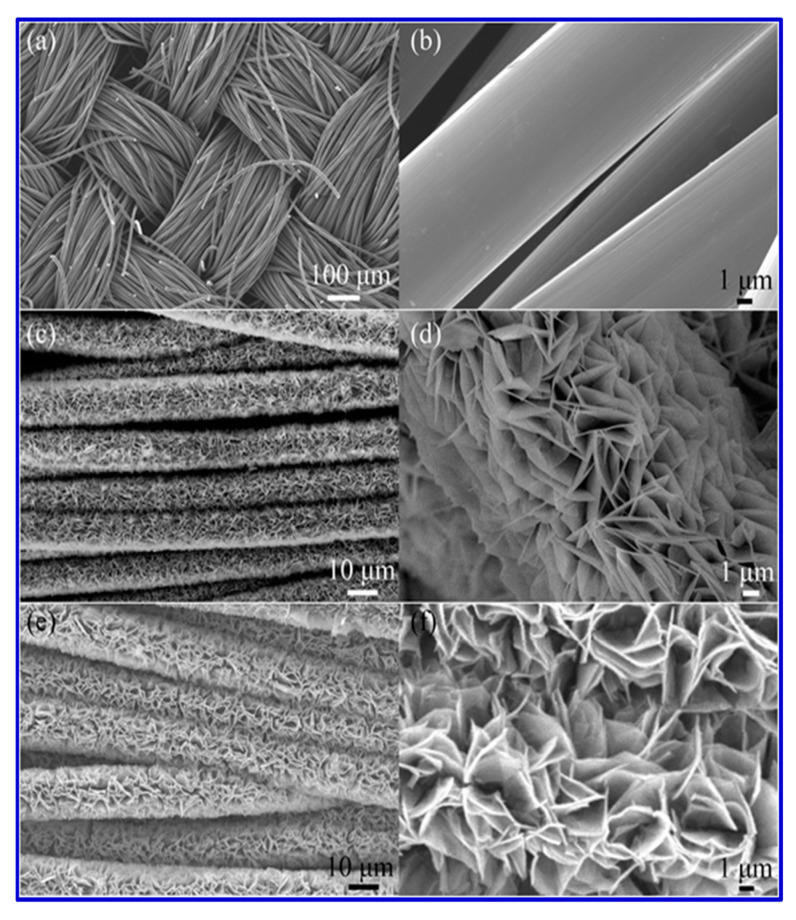
(**a**,**b**) SEM images of the carbon cloth; (**c**,**d**) NiO grown on the carbon cloth; and (**e**,**f**) hybrid nanostructures of carbon cloth integrated with NiO@MnO_2_ (reproduced with permission from ref. [111]).

Among various metal oxides, NiO has been widely investigated for SC applications. Ramesh et al. recently reported the synthesis of NiO/MnO_2_@N-doped graphene oxide for SC electrode material [110,111]. The composite was nanocrystalline in nature with a large surface area and facilitated ion/electron transport. The composite offered a specific capacitance of 1490 F/g at a current density of 0.5 A/g. The synergistic effect of NiO@MnO_2_ oxides over the graphitic sheets was believed to result in the very high capacitance. Xi et al. reported NiO/MnO_2_ core–shell nanoflakes over a carbon cloth for flexible SC electrodes [111], which is essential for further development of flexible and foldable electronic gadgets. They grew NiO on the carbon cloth using a hydrothermal process and then an MnO_2_ thin film covered the NiO structure by the self-limiting process. Field emission scanning electron microscopy (FESEM) images (Figure 4) of the fabricated NiO-coated MnO_2_ showed beautiful flower petal morphology that is a signature of high surface area of the developed prototype materials for hybrid SCs [110,111]. The NiO nanosheets were grown perpendicularly over the carbon fibers, which led to a highly porous structure beneficial for the diffusion of electrolyte into the electrode [112].

**Figure 5:**
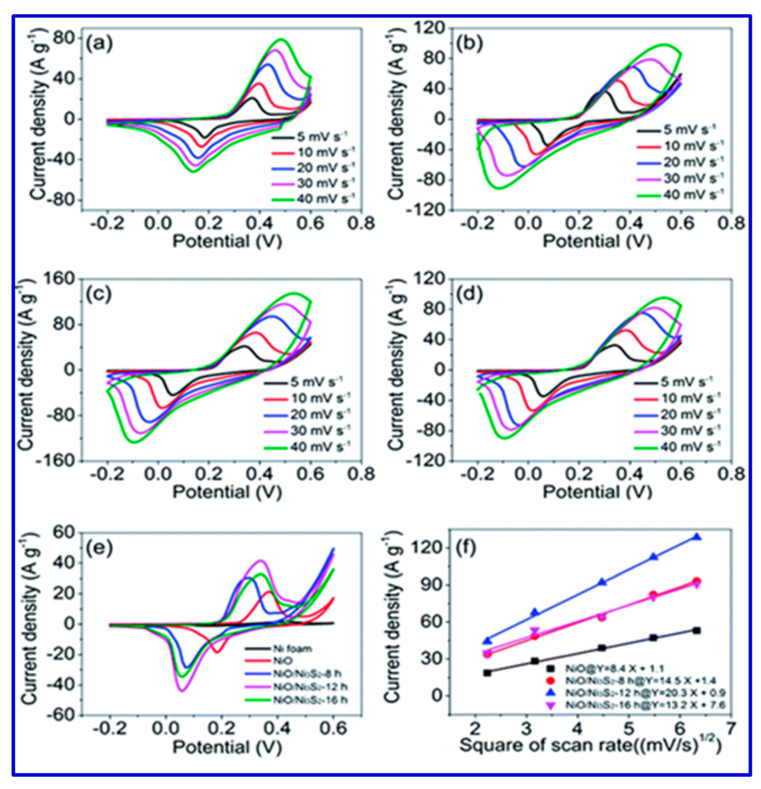
Cyclic voltammetry (CV) plots of (**a**) NiO, (**b**) NiO/Ni_3_S_2_-8 h, (**c**) NiO/Ni_3_S_2_-12 h, and (**d**) NiO/Ni_3_S_2_-16 h (h denotes sulfuration time) electrodes at different scan rates. (**e**) Comparative study of NiO, NiO/Ni_3_S_2_-8 h, NiO/Ni_3_S_2_-12 h, and NiO/Ni_3_S_2_-16 h electrodes at a scan rate of 5 mV s^−1^. (**f**) Linear relationship between cathodic peak current and square root of scan rate (reproduced with permission from ref. [112]).

It was observed that upon further deposition of the MnO_2_ layer, the morphology was retained, and the results revealed that the hybrid structure was highly integrated. Such nanoflakes exhibited an aerial specific capacitance of 316.37 mFcm^−2^ with a Coulombic efficiency of > 97%. The as-synthesized binder-free electrode possessed superior electrochemical behavior for flexible SCs. Liu et al. reported tunable sulfuration engineered electrodes made from NiO/Ni_3_S_2_ nanosheets constructed over a porous Ni foam and achieved a specific capacitance of 2153 F/g [112]. They constructed an asymmetric SC device using the NiO/Ni_3_S_2_ nanosheets as the positive electrode, activated carbon as the negative electrode, and 3 M KOH as the electrolyte, and the device performance was evaluated by cyclic voltammetry (CV) study [109,110,111]. Liu et al. considered that the redox reactions were the main reason for the good performance, and the CV features of their testing results are shown in Figure 5.

**Figure 6:**
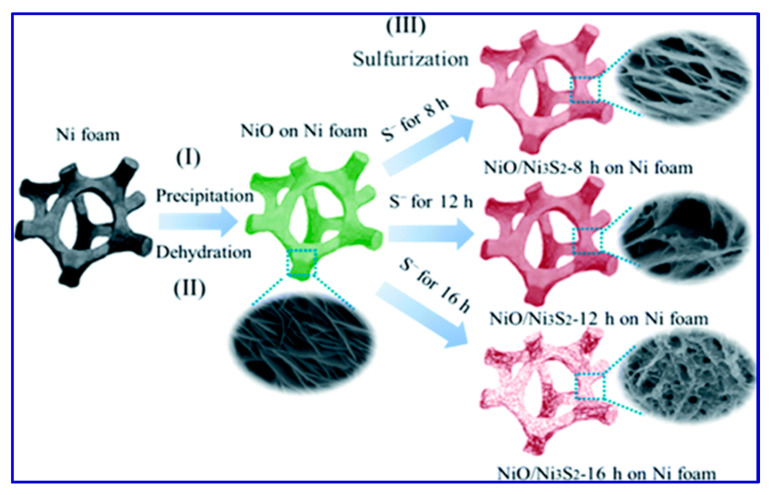
Schematic for the synthesis of foam-like nanocomposites using NiO/Ni_3_S_2_ nanosheets over Ni foam by different approaches (reproduced with permission from ref. [112]).

From Figure 5, Liu et al. concluded that a linear dependence between the cathodic peak current and the square root of the scan rate presented in the redox reaction at the electrode/electrolyte interface was caused by the diffusion-controlled non-surface processes. They also suggested that the sulfuration treatment played a vital role in the fast redox reactions, thereby increasing the capacitive behavior [112]. A schematic of the synthesis of electrode material for SCs using NiO/Ni_3_S_2_ nanosheets over Ni foam is presented in Figure 6.

**Figure 7:**
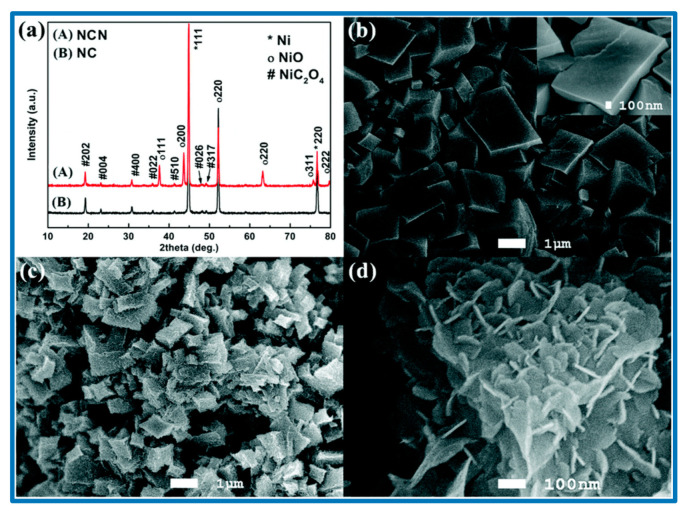
(**a**) XRD patterns of the nanocomposites; (**b**) SEM image of the Ni foam@NiC_2_O_4_; and (**c**,**d**) SEM images of the NiC_2_O_4_@NiO core–shell structure (reproduced with permission from ref. [113]).

From the schematic, it is clear that the morphology of the resulting nanostructures depends on the sulfuration reaction time (8 h, 12 h, and 16 h), as presented in Figure 6. He et al. synthesized another electrode material for SCs over Ni foam, namely tremella-like NiC_2_O_4_@NiO core/shell nanostructures, which offered a specific capacitance of 2287 F/g at a current density of 1 A/g [113]. They demonstrated that such tremella-like morphology (Figure 7) increased the number of active sites for the redox reactions, which enabled the effective penetration of the electrolyte, shortened the diffusion pathway, and thereby increased the conductivity [114]. He et al. studied the structural and morphological changes of the fabricated samples using powder X-ray diffraction (XRD) technique (Figure 7), and a distinct variation in the diffraction patterns was observed, which is in good accordance with the morphological features. To evaluate the electron-hopping process on the surface of materials that is the backbone of charge accumulation and redox reactions, electrochemical analyses were carried out in 2 M KOH solution. All the CV curves depicted the well-defined redox peaks, indicating the pseudocapacitive behavior of the nanocomposites [113]. In an alkaline solution, the faradaic reaction occurred according to the following equation:

$$\mathrm{NiO}+{\mathrm{OH}}^{-}\leftarrow ------\to \mathrm{NiOOH}+e^{-}$$

Thus, due to the extra electrons formed, the heterostructure electrode exhibited a high specific capacitance, with a good rate capability and cyclic stability. After 10,000 cycles, the capacitance remained at 95% of the initial value, which is very impressive.

This pioneering work inspired many researchers to further test analogue materials for their applicability in SCs. Within 4 years (2015–2018), a huge number of works on tremella-like core/shell nanostructures using different metals were published, and some highly efficient hybrid materials have been identified for SCs [111,112]. However, the selection of an electrolyte for these electrode materials demands further optimization, because a poor combination with an electrolyte could create problems, not only for efficiency but for future commercialization as well [112,113].

In this regard, several metal sulfides have attracted special attention due to their easy synthesis [113,114]. Among many of them, nanosized NiS is a typical pseudocapacitive electrode material for SCs. Its salient features, such as high redox activity, good capacitive performance, and ease of processability, make it particularly attractive [114]. Guan et al. reported the synthesis of NiS micro-flowers, with improved surface area and enhanced electron transfer rates, the electrode made of these flowers exhibited high capacitive performance, offering a specific capacitance of 1122.7 F/g at a current density of 1 A/g [114]. Such a hierarchical structure facilitated easy access of its surface to the electrolyte. The electrochemical activity in 3 M KOH electrolyte showed faradaic reactions according to the following equation:

$$\mathrm{NiS}+{\mathrm{OH}}^{-}\leftarrow ------\to \mathrm{NiOH}+e^{-}.$$

**Figure 8:**
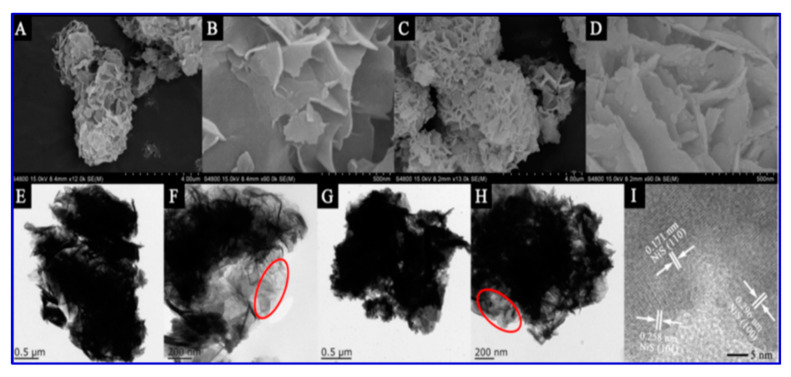
SEM images of (**A**,**B**) precursor; (**C**,**D**) NiS micro-flowers; (**E**,**F**) TEM images of the precursor; (**G**,**H**) NiS micro-flowers, and (**I**) HRTEM lattice image of the NiS flowers (reproduced with permission from ref. [114]).

An asymmetric capacitor, using the micro-flowers as the positive electrode against an activated carbon negative electrode, delivered an energy density of 31 Wh/kg with a power density of 0.9 kW/kg. In addition, the asymmetric capacitor showed capacitance retention of 114.1% at 5 A/g. The morphology of the nanostructured NiS was characterized by SEM and transmission electron microscopy (TEM), and the results are presented in Figure 8. The flower-like morphology depicts the porous structure and high surface area in the SEM images, while the dark spots in the TEM images show the heterogeneous structure along with a homogeneous coating of NiS [114].

**Figure 9:**
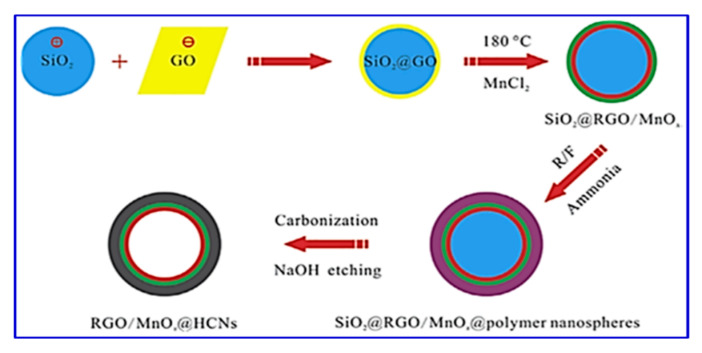
Schematic of the synthesis of core–shell rGO/MnO_2_@hollow carbon nanospheres (reproduced with permission from ref. [115]).

Owing to its abundance, low cost, and high specific capacitance feature, Mn_3_O_4_ has also been explored as an electrode material for SC applications. Xiong et al. prepared Mn_3_O_4_ nanoparticles and incorporated them in rGO films, aiming to develop flexible electrodes [93]. Their asymmetric SCs showed a volumetric capacitance of 52.5 Fcm^−3^ at 0.2 Acm^−3^ in a Na_2_SO_4_ electrolyte [93,94]. The intercalation of Mn_3_O_4_ nanoparticles into the rGO paper improved the conductive behavior of the rGO. Surface morphology studies revealed that the nanoparticles were uniformly distributed between the rGO layers, which resulted in efficient charge transfer and reduced the restacking issues of the rGO sheets [93]. Meanwhile, the rGO sheets also acted as frameworks to support the Mn_3_O_4_ nanoparticles and prevented them from dissolution and aggregation (Figure 9). Such a synergistic effect between the two components led to the formation of an easy and continuous ion-transport network with enhanced rate kinetics and stability [93].

**Figure 10:**
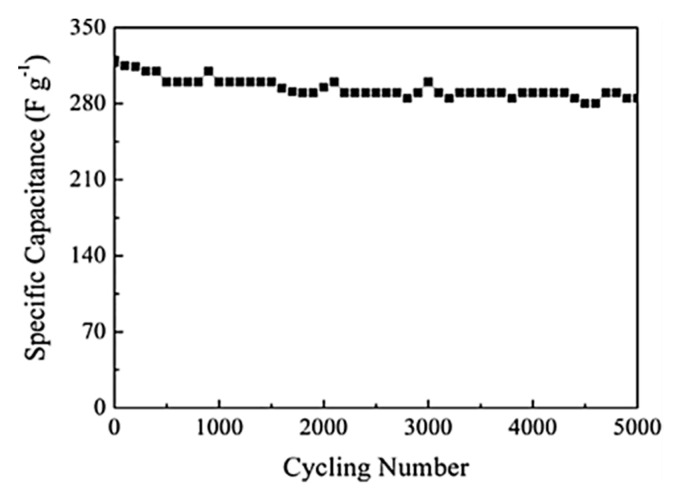
Cyclic performance of electrode material rGO/MnOx@HCN_0.4_ at a current density of 0.5 A/g in 6 M KOH solution (reproduced with permission from ref. [115]).

Liu et al. reported a similar rGO/MnOx@carbon hollow nanosphere (HCNS) core–shell structure for application in SC electrodes [115]. The method for the preparation of these nanocomposites is depicted in Figure 9. They have proposed that the Mn^2+^ bind with the negatively charged O^2−^ of the graphene oxide, which leads to a 3D core–shell structure offering good electrochemical behavior with an 88% capacitance retention after 5000 cycles (Figure 10).

**Figure 11:**
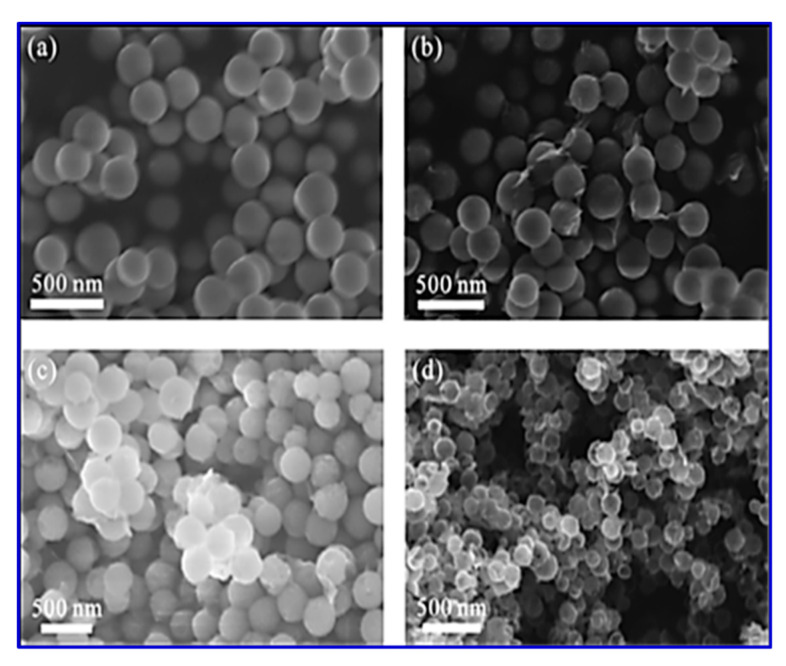
SEM micrographs showing the morphology and surface roughness. (**a**) SiO_2_, (**b**) SiO_2_@GO, (**c**) SiO_2_@rGO/MnO_x_, and (**d**) rGO/MnO_x_@HCNs (reproduced with permission from ref. [115]).

The composite possesses a hollow geometry with a uniform outer shell of 10 nm and inner spherical pores of 150 nm. CV studies were carried out in a two-electrode system in a 6 M KOH electrolyte. The electrode material showed an energy density of 9.38 Wh/kg and a power density of 500 W/kg at a current density of 1 A/g. The material also offered a high rate capability with a specific capacitance of 250 F/g in the two-electrode configuration [115]. Thus, the new nano-architecture highlights a great potential for core–shell electrode materials in SCs. The surface morphology of all the components used in their study is shown in Figure 11. The SEM morphology provides clear information regarding the internal structure of these nano- and macro-materials. It is interesting to note that the spherical shape remains in every sample (viz. (a) SiO_2_, SiO_2_@GO, SiO_2_@RGO/MnO*_x_*, and RGO/MnO*_x_*@HCNs).

**Figure 12:**
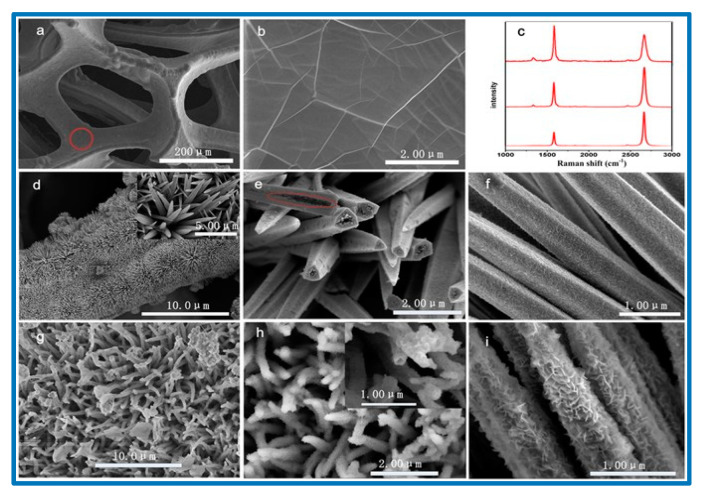
(**a**) SEM image of graphene nanoflake (GNF), (**b**) high-magnification SEM image of graphene encircled in an image, (**a**,**c**) Raman spectra of graphene grown over Ni foam, (**d**) GNF/NiCo_2_S_4_ nanotube (NCS)-precursor on 3D Ni foam, (**e**,**f**) GNF/NCS on second hydrothermal treatment, and (**g**–**i**) GNF/NCS/CNS core–shell 3D architecture (reproduced with permission from ref. [116]).

Another porous nanostructured material for SC application was reported by Beka et al. [116]. They synthesized a nickel-cobalt sulfide (NiCo_2_S_4_) core–shell structure on a 3D graphene framework and consequently evaluated its electrochemical performance. In their synthesis, a NiCo_2_S_4_ nanotube (NCS) acted as the core and Co*_x_*Ni_(3−*x*)_ S_2_ (CNS) nanosheets as the shell. The 3D graphene was first grown over the Ni foam using a chemical vapor deposition process, named graphene nanoflake (GNF), and then the NCS/CNS was grown on the 3D graphene using a hydrothermal process. The high mechanical stability of 3D graphene provided excellent support for the entire system, and the high conductivity of the graphene network offered superb transport channels between the collector and active material [116]. The uniform porous forest became a 3D architecture with more surface-active sites after the core/shell formation, thus leading to increased charge storage [116]. Figure 12 shows the surface morphology of the reported electrode materials.

**Figure 13:**
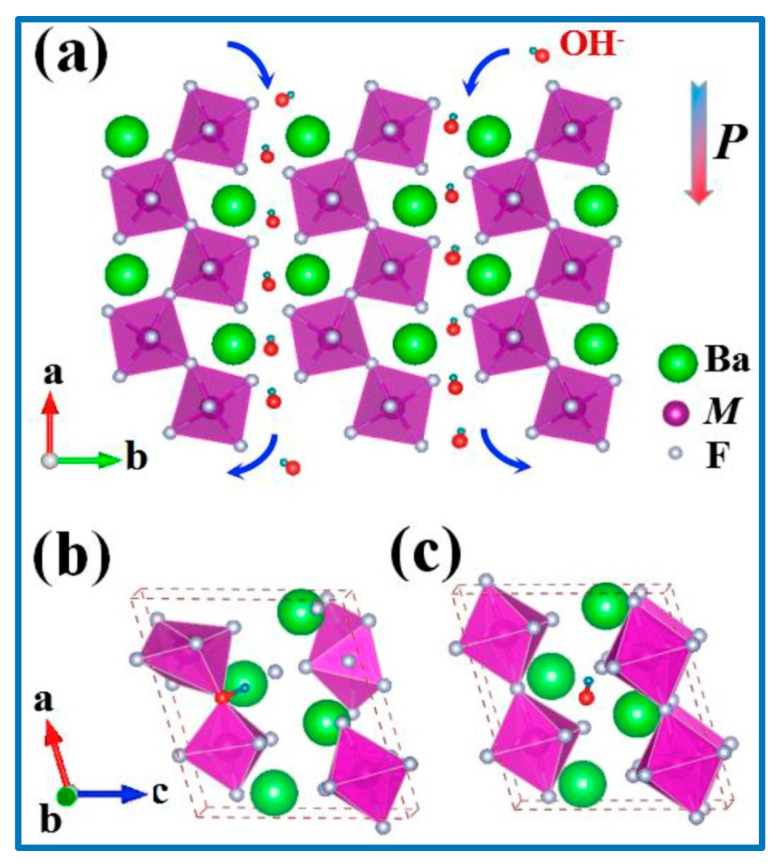
Schematics of (**a**) migration of the OH^−^ ions between layer space of BaMF_4_, (**b**) BaMF_4_+OH after the density-functional theory (DFT) optimization with M-O-M bonding, and (**c**) BaMF_4_^+^OH after DFT optimization for non-bonding (or weak bonding) between O and M atoms (reproduced with permission from ref. [110]).

The graphene layer sandwiched between the NCS/CNS core–shell and the nickel foam current collector aided the superb electron transport and led to an aerial capacitance of 15.6 F cm^−2^ at a current density of 10 mA cm^−2^, with cyclic stability of 93% after 5000 cycles and a rate capability of 74.36% [116]. Zhou et al. reported a new hybrid material with layered porous structure as SC electrodes [110]. They studied the performance of a new series of layered barium transition-metal fluorides, BaMF4 (M = Mn, Co, and Ni). In the layered structure, spaces existed between layers, leading to the formation of numerous interfaces [110]. The interlayer spaces acted as a reservoir for the anions, which could be driven in and out depending on the externally applied electric field or inner built electric field. Figure 13 shows the arrangement of the metal atoms in the lattice structure. In the density-functional theory (DFT) investigation it was found that among the three studied electrode systems, M ¼ Co showed the largest capacitance, best conductivity, and cycle stability. Considering the very early stage of using BaMF4 as electrodes, we believe that there is enough space in the near future to greatly improve their SC performance [110,117].

**Figure 14:**
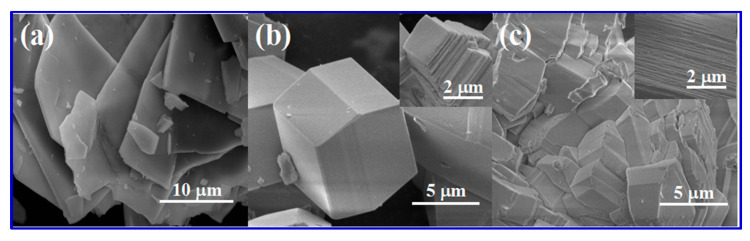
SEM images showing the morphology of the materials as theoretically predicted by Zhou et al., as presented in Figure 15 [110]. (**a**) Pure BaMnF_4,_ (**b**) pure BaCoF_4_, and (**c**) pure BaNiF_4_ (reproduced with permission from ref. [115]).

Zhou et al. further compared the theoretical results with experimental results. The morphology of the studied nanomaterials ((a) BaMnF4, (b) BaCoF_4_, and (c) BaNiF_4_) is shown in Figure 14. Interestingly, the shapes of the crystals appearing in the SEM micrographs were amazingly similar to the results shown in Figure 13. The SEM morphology (Figure 14) of the bulk materials (BaCoF4 and BaNiF4) was not suitable for application as SC electrode materials, owing to very low surface area and lack of porosity. These SEM images agreed well to the theoretical results, as obtained by Zhou et al. [110]. However, its exfoliation under suitable conditions produced an ideal nanostructure that was suitable for the SC applications.

**Figure 15:**
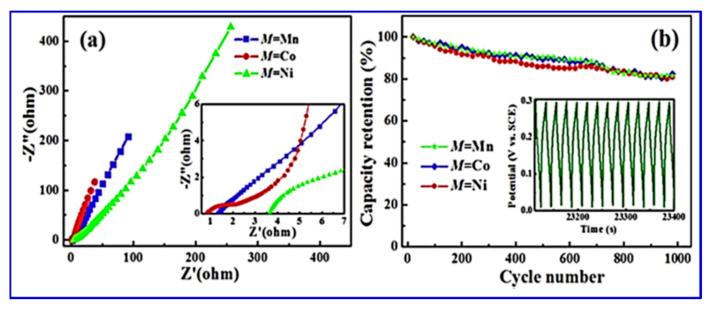
(**a**) Nyquist plots of BaMF_4_ electrode with different M centers. (**b**) Cyclic performance of the three BaMF_4_ electrodes. Inset: galvanostatic charge/discharge (GCD) cycling of BaCoF_4_ electrode (reproduced with permission from ref. [115]).

The surface morphology analyses, as shown in Figure 14, revealed that a layered structure with a stratified structure was formed, due to the anisotropic crystal growth under hydrothermal conditions, which resulted in a quasi-2D crystalline structure. The puckered sheets can provide free space with the increased surface area for fast ion diffusion, which benefits the electrode performance. The valence-variable metal ions led to improved faradaic redox reactions at the electrode interface, which contributed to the electrochemical performance of the electrode material. CV and electrochemical impedance spectroscopy (EIS) studies in a three-electrode system with 6 M KOH electrolyte depicted the specific capacity and specific capacitance of BaMF_4_ by varying the M centers with Mn, Co, and Ni. The EIS study (Figure 15) also revealed the equivalent series resistance of the respective systems. Table 6 tabulates the data corresponding to the different metal centers [110,117].

**Figure 16:**
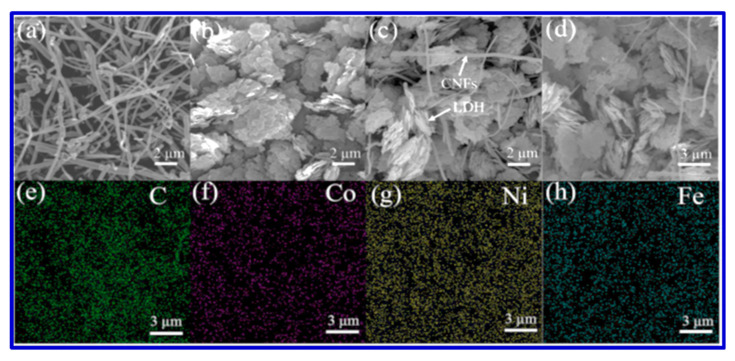
SEM images of (**a**) carbon nanofibers (CNFs_, (**b**) CoNiFe-LDH, and (**c**) CoNiFe-LDH/CNFs-0.5 composite, and (**d**–**h**) SEM image and elemental distribution maps of C, Co, Ni, and Fe of CoNiFe-LDH/CNFs-0.5 composite (reproduced with permission from ref. [17]).

**Figure 17:**
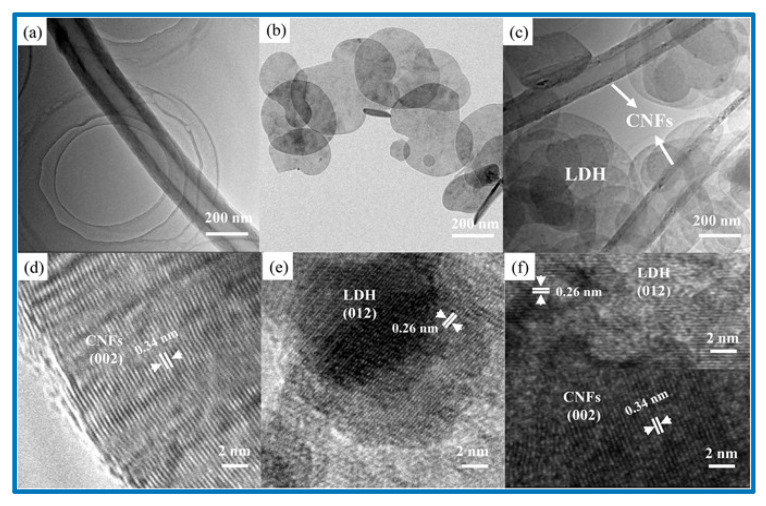
(**a**–**c**) TEM and (**d**–**f**) HRTEM images of CNF-based electrode materials for SCs. (**a**,**d**) CNFs, (**b**,**e**) CoNiFe-LDH, and (**c**,**f**) CoNiFe-LDH/CNFs-0.5 composite (reproduced with permission from ref. [17]).

Layered double hydroxides (LDHs) have been extensively cultivated as a potential pseudocapacitive material, since their high specific capacitance, good redox reversibility, and excellent ion-exchange property [17]. Compared with binary-component LDH, ternary-component LDH has much better electrochemical activity because of an increased number of active sites after incorporation of the third metal ion. Such a ternary-component LDH has been studied by Wang et al., who reported the synthesis of CoNiFe-LDH/carbon nanofibers (CNFs) [17]. The composite CoNiFe-LDH/CNFs displayed a high specific capacitance of 1203 F/g at a current density of 1 A/g and an excellent long-term cyclic stability of 94.4% after 1000 cycles. Wang et al. also constructed an asymmetric supercapacitor with CoNiFe-LDH/CNFs as the positive electrode and activated carbon as the negative electrode. Their device offered a specific capacitance of 84.9 F/g at a current density of 1 A/g and an energy density of 30.2 Wh/kg [17]. The SEM images of the original CNFs in Figure 16 show the entangled-network structure with uniform dispersion. The SEM image of CoNiFe-LDH also presents the formation of irregular nanosheets. After the incorporation of CNFs, the aggregated structure in Figure 16b is somewhat reduced, as shown in Figure 16c,d, and such a structure was beneficial for faster ion diffusion and electronic transportation. Using elemental mapping, Wang et al. also proved the presence of three metal centers in the composite. The further detailed structure of the hollow tubular CNFs was also unveiled using TEM images (Figure 17) [17]. Based on these HRTEM and SEM images, Wang et al. claimed that they fabricated the nanocomposite with high surface areas, which contributed to the excellent performance [17].

**Figure 18:**
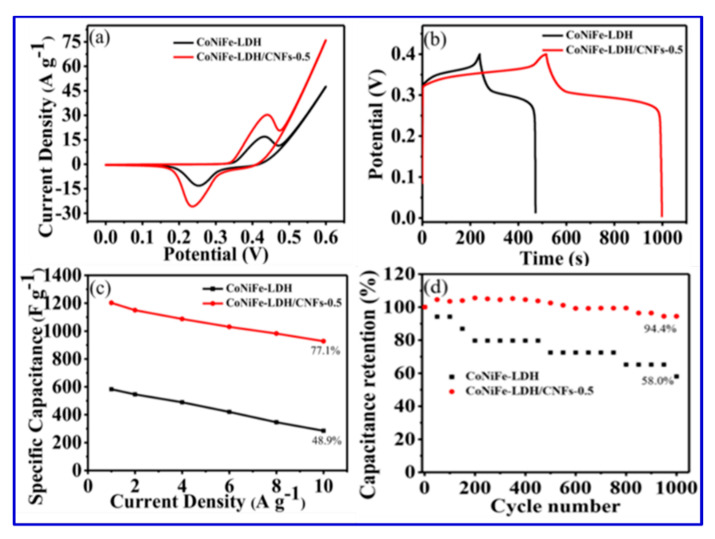
(**a**) CV graphs of CoNiFe-LDH and CoNiFe-LDH/CNFs-0.5 at 5 mV s^−1^. (**b**) GCD curves of CoNiFe-LDH and CoNiFe-LDH/CNFs-0.5. (**c**) Specific capacitance of CoNiFe-LDH and CoNiFe-LDH/CNFs-0.5 composite at varying current densities. (**d**) Specific capacitance retention (%) of CoNiFe-LDH/CNFs-0.5 composite at a current density of 20 A/g and CoNiFe-LDH at a current density of 10 A/g (reproduced with permission from ref. [17]).

Indeed, by incorporating CNFs into the double-layer hydroxide, the rate capability and specific capacitance of the composite were significantly improved, consistent with their CV results, as shown in Figure 18.

**Figure 19:**
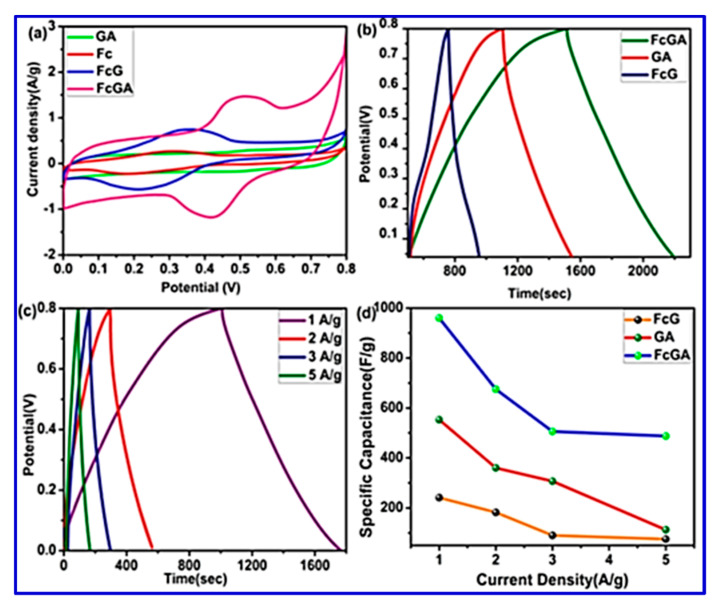
(**a**) CV graphs of nanocomposites at 1 mV s^−1^, (**b**) GCD plots of composites, (**c**) galvanostatic charging-discharging plot of FcGA at different current densities, and (**d**) variation of specific capacitance at different current densities of nanocomposites (reproduced with permission from ref. [106]).

The FESEM study of the synthesized composite revealed a network-like intertwined structure, which can be effective for enhancing the surface area, thereby leading to easy accessibility of the electrolyte ions. BET surface-area analysis has also demonstrated that FcGA exhibited a maximum surface area of 231 m^2^g^−1^, which could also influence the electrochemical performance. The electrochemical analysis was carried out in 1 M NEt_4_BF_4_-acetonitrile solution as depicted in Figure 19, and the electrodes gave a high specific capacitance of 960 F/g and an energy density of 76.44 Wh/kg at a current density of 1 A/g.

**Figure 20:**
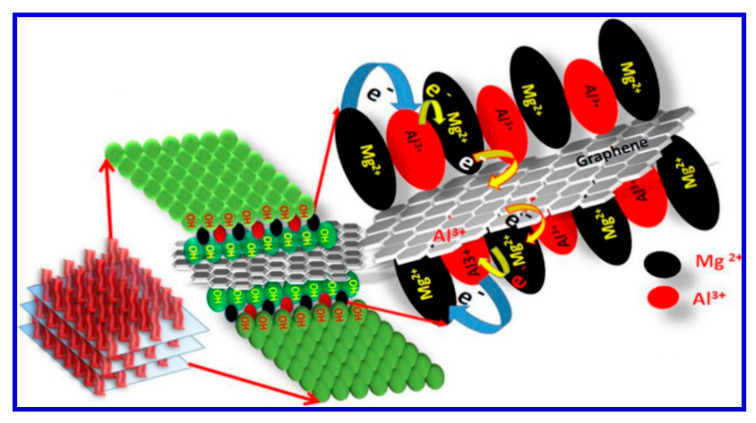
Mechanism of electron transfer (charge transfer from LDH to rGO via electron hopping) in the electrode materials for SC applications developed and tested by Hatui and coworkers (reproduced with permission from ref. [121]).

In recent years, double-hydroxide-based SCs have attracted significant attention, and several outstanding works on the subject have been published [118,119,120]. In this regard, by advancing the fabrication of graphene-based composites as electrode materials for SC applications, our research group achieved an Mg-Al LDH using rGO [121]. Utilizing a one-pot synthesis approach, we first produced sandwich-like Mg/Al LDH anchored with rGO to form the composite. Hatui et al. explained that the exceptional efficiency of these electrode materials was due to the unique manner of electron transfer from metallic layers to rGO. The process of electron transfer from one phase to the other according to Hatui and coworkers was schematically presented in Figure 20 [121].

**Figure 21:**
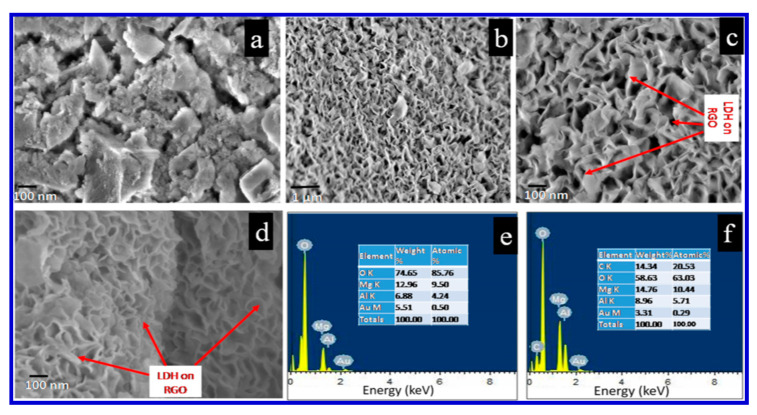
FESEM images of (**a**) pristine MgAl LDH nanoparticles, (**b**) rGO@MgAl LDH at low magnification, (**c**) rGO@MgAl LDH at higher magnification (flower-petal-like structure, top view), (**d**) rGO@MgAl LDH at higher magnification (side view), (**e**) EDS spectrum of MgAl LDH, and (**f**) EDS spectrum of MgAl LDH (reproduced with permission from ref. [121]).

Hatui et al. used a variety of techniques, including Fourier-transform infrared (FTIR) spectroscopy, X-ray photoelectron spectroscopy (XPS), and XRD analysis along with FESEM, TEM, and atomic-force microscopy (AFM), to verify the sandwiched structure [121]. It has been found that the special sandwiched morphology of the newly developed rGO-based Mg/Al LDH played a very crucial role in achieving maximum performance [121]. Selected FESEM images and energy-dispersive X-ray (EDX) graphs of such samples are exhibited in Figure 21.

Hatui et al. also carried out electrochemical analyses and characterizations of their composites in 1 M aqueous KOH, which gave rise to a specific capacitance of 1334 F/g at a current density of 1 A/g in a three-electrode system [121]. To avoid errors, they also tested the efficiency of developed materials with a two-electrode cell system and recorded a specific capacitance of 1092.5 F/g in 1 M TEABF4 (in acetonitrile solution) as the electrolyte at a current density of 2 A/g. They further reported cyclic stability of approximately 87% retention of specific capacitance after 10,000 consecutive charge–discharge cycles at a steady current density of 5 A/g for the two-electrode organic electrolyte system. The energy density of the nanocomposite also shows a high value of 388.26 Wh/kg at a current density of 2 A/g and a power density of 3198.48 W/kg in the two-electrode organic electrolyte system. The importance advance of this DLH material over others is mainly down to the very simple, one-pot solvothermal technique, which is the cheapest and most facile route for the fabrication of sandwich-like RGO@ MgAl LDH nanocomposites. Moreover, this method also offered a simultaneous reduction of GO to rGO, and therefore the growth of MgAl LDH could greatly enhance both the conductivity of the entire system and reduce the internal resistance, which, in turn, may improve the specific capacitance due to the very high surface area of the nanocomposites [121]. Thus the discussions of various studies carried out by different research groups in recent years confirm the wide applicability of SCs in future energy-storage devices. The SCs can supply quick bursts of power for consumer electronics and vehicles, thus help to reduce the need for non-renewable energy sources, eventually contributing to reduce their depletion.

We appreciate that the work reported by Hatui et al. adopted a very easy and facile route for the synthesis of LDH-based graphene interlinked nanocomposites for prototypical SC applications. The proposed mechanism for the electron hopping from the LDH to rGO in the electrode materials for the SC applications was diagrammatically presented in Figure 20. It is interesting to note that the newly developed LDH using rGO showed a much greater efficiency than those reported previously, although Hatui et al. adopted a very simple and green method for the materials fabrication [122,123,124,125,126]. Some recently developed LDH works are summarized in Table 7 for further reading.

**Figure 22:**
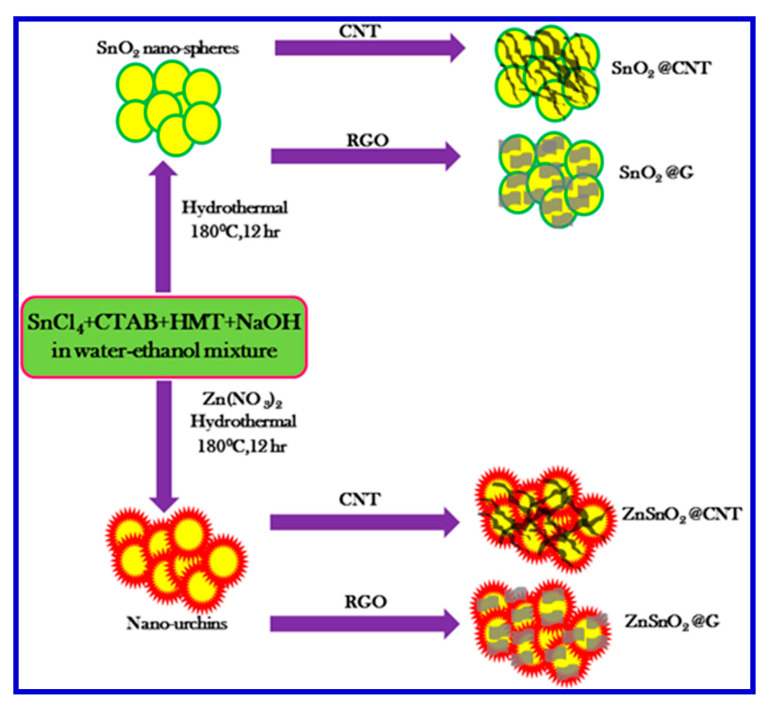
Representation of synthesized hierarchical Zn-doped SnO_2_ nano-urchins decorated on rGO nanosheets (reproduced with permission from ref. [130]).

Amrita and coworkers developed a very smart rGO-based nanomaterial, involving the fabrication of hierarchical Zn-doped SnO_2_ nano-urchins decorated on the rGO nanosheets (Figure 22) as electrode materials for SCs [130]. The composite electrode offered a specific capacitance of 635 F/g at a current density of 1 A/g and had high cyclic stability up to 5000 cycles with a capacitance retention of 78.4% [130]. They believed that the excellent energy storage capacity originated from the Zn-doped SnO_2_ nanospheres in the presence of rGO, which tailored the morphology and the electrical properties [24]. They explained that Zn^2+^ doping in the SnO_2_ nanospheres prevented Sn clustering, thereby reducing the particle size, which led to the formation of urchin-like nanostructures. These urchins with a high surface area and short transport paths can offer high capacitive performance through assembly with rGO nanosheets. Amrita and coworkers used XPS and XRD techniques to confirm the presence of SnO_2_ crystal planes and the successful Zn^2+^ doping. The incorporation of the rGO nanosheets augmented the Coulombic efficiency, specific capacitance, and cyclic performance. The enhancement of the capacitive behavior was attributed to the synergistic effects of the pseudocapacitance and double-layer capacitance.

**Figure 23:**
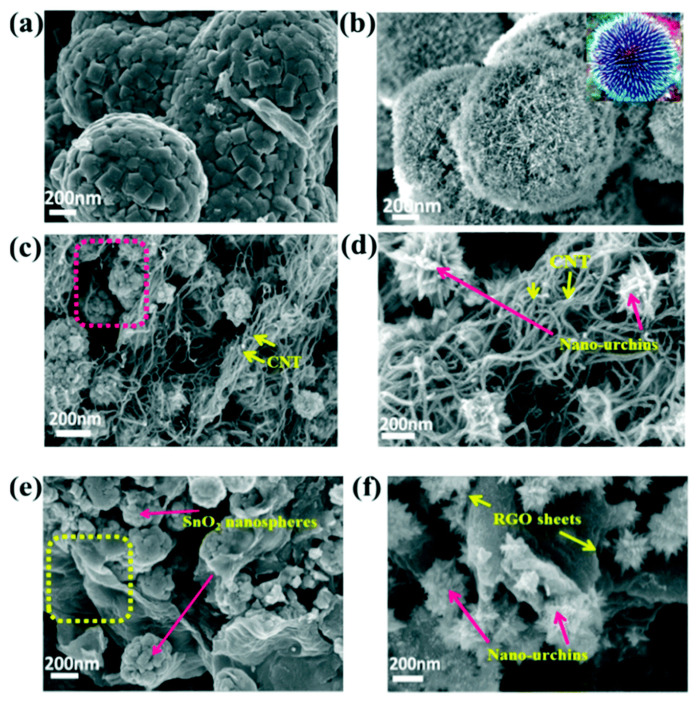
FESEM images of different nanocomposites. (**a**) SnO_2_ nanospheres, (**b**) ZnSnO_2_ nano-urchins (inset showing a sea-urchin structure), (**c**) SnO_2_@CNT, (**d**) ZnSnO_2_@CNT, (**e**) SnO_2_@G, and (**f**) ZnSnO_2_@G (reproduced with permission from ref. [130]).

Amrita et al. evaluated the surface morphology of their nanocomposites using FESEM, and the results were shown in Figure 23a, in which the SnO_2_ nanospheres exhibited a very uniform spherical structure [130]. Upon doping the SnO_2_ with Zn^2+^, a clear spike-like morphology was observed, which resembles a sea urchin structure (inset of Figure 23a–f). Furthermore, with the addition of CNTs into SnO_2_ and Zn-doped SnO_2_, as named as SnO2@CNT and ZnSnO_2_@CNT for comparison, shown in Figure 23c,d, the nanospheres and nano-urchins were found nicely intertwined within the CNTs. Upon the addition of CNTs, the nanospheres and nano-urchins were well separated from each other, having condensed size. This configuration resulted in a very high surface area and uniform distribution. By incorporating rGO further with these nanomaterials, as SnO_2_@G and ZnSnO_2_@G, the nanospheres were also found wrapped by the rGO nanosheets, as revealed in Figure 23e,f. The yellow surrounded area in Figure 23e depicts the presence of rGO sheets inside which the SnO_2_ nanospheres are integrated. Figure 23f also shows the uniform and good distribution of the nano-urchins over the rGO. The yellow area indicates the presence of rGO nanosheets within the nano-urchin structures. Further magnified FESEM micrograph of the ZnSnO_2_@G illustrates the fine dissemination of nano-urchins over rGO, as arrowed in Figure 23f. These vivid surface morphological studies on these nanocomposites have revealed the effective assembly of the nano-urchins over the rGO or CNTs, as well as the effect of Zn^2+^ doping.

Prior to Amrita et al., Li and their coworker have developed a facile approach to fabricate three-dimensional ZnO-rGO-ZnO sandwich-structures by incorporating ZnO powder into the reaction of graphitic oxide, followed by heating until the proper reduction occurred [100]. In this process, fine ZnO nanorod arrays with the size of 20–40 nm grew on both surfaces of rGO nanosheets unswervingly. Compared with plain rGO, the as-synthesized ZnO/rGO/ZnO nanocomposites displayed higher specific surface areas [100]. The electrochemical behavior of ZnO/rGO/ZnO nanocomposites electrodes obtained the highest specific capacitance of 275 F/g at a scan rate of 5 mVs^−1^ in 1.0 M Na_2_SO_4_ electrolyte, by chronopotentiometry. However, according to authors, the repeatability is essential to be double-checked and further study is needed.

They also showed that the as-fabricated hybrid ZnO/rGO/ZnO sandwich-structures exhibited an excellent rate capability and outstanding long-term cycling stability, as compared with the pure rGO and pristine rGO. This excellent work of Li and the coworker established that this ZnO/rGO/ZnO nanostructure is an auspicious candidate as electrode material for high-performance SCs [100].

**Figure 24:**
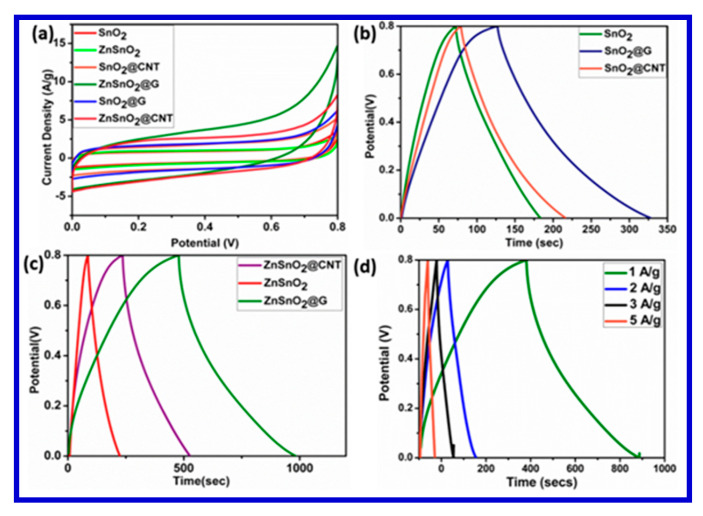
Electrochemical performance of the electrode materials. (**a**) CV at a scan rate of 10 mVs^−1^; (**b**,**c**) galvanostatic charging–discharging of the nanocomposites; and (**d**) galvanostatic charging–discharging of the ZnSnO2@G nanocomposite at different current densities (reproduced with permission from ref. [130]).

**Figure 25:**
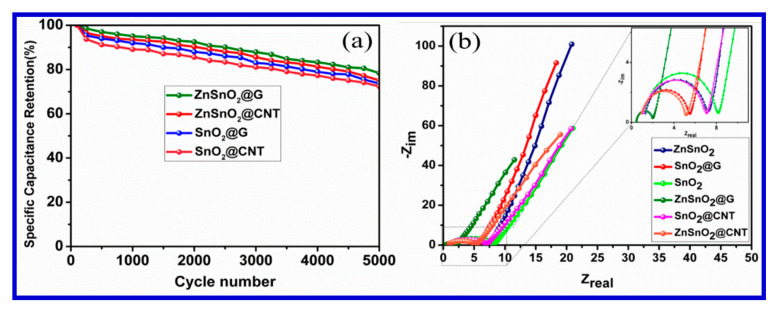
(**a**) Specific capacitance retention (%), and (**b**) Nyquist plots of the ZnSnO2@G nanocomposites (reproduced with permission from ref. [130]).

To evaluate the electrochemical performance of these materials as a working electrode, Amrita et al. conducted CV and galvanostatic charging–discharging analyses in an aqueous 1 M KCl electrode, in a potential window of 0–0.8 V using a three-electrode system. Figure 24 shows the CV curves of all nanocomposites at a scan rate of 10 mVs^−1^. The CV curves all depict a quasi-rectangular nature without any obvious peak, indicating the typical behavior of the EDLCs. The symmetric quasi-rectangular forms and an increased current density indicate the fast and reversible faradaic reactions and perfect capacitive behavior. A slower voltage scan at 10 mVs^−1^ was carried out to reduce the flux at the electrode surface compared with higher scan rates. This can be attributed to the fact that as the current is directly proportional to the flux, the magnitude of the current should be high at faster scan rates and low at slower scan rates. The results of the electrochemical analyses for these electrode materials are presented in Figure 25a,b [130]. In addition, the cyclic stability of their materials was tested up the 5000 cycles, and the results are presented in terms of Nyquist-plot specific capacitance retention parameters (Figure 24).

In addition to the above SnO_2_-based electrode materials, many other types of materials, particularly MoS_2_, an analogue of 2D graphene and its derivatives, have been intensively investigated by different groups [130,131,132,133,134,135,136,137,138]. These emerging materials have shown great potentials for practical applications. The layered MoS_2_ nanostructures can contribute to the double-layer capacitance from their interlayer charge storage, in addition to their intralayer charge-storage, thus becoming an ideal candidate as SC electrode materials. Similar to the much-studied RuO_2_, MoS_2_ may also provide an added capacitance from the faradaic reaction at the Mo metal center, owing to its multiple and tunable oxidation states varying from 2^+^ to 6^+^ [133,134,135,136,137]. Furthermore, the 2D layered nanostructure of MoS_2_ provides excellent electron hopping, and greater ionic conductivity, compared with its corresponding oxides for the same purposes.

**Figure 26:**
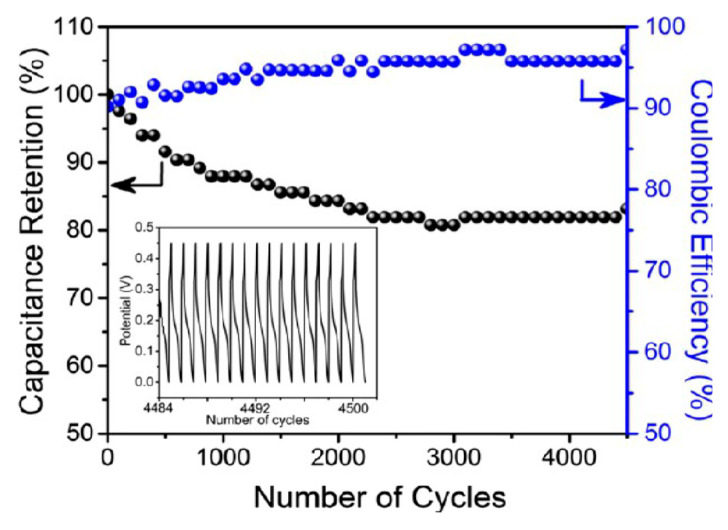
Cyclic stability as revealed by capacitance retention and corresponding charging electrode (CE) for MoS2@3D-Ni-foam grown by atomic layer deposition after 4500 cycles (inset showing the last several charge–discharge cycles; reproduced with permission from ref. [138]).

Very recently, Nandi et al. reported a direct growth of MoS_2_ on a 2D stainless-steel foil surface and on a 3D Ni foam by a plasma-enhanced atomic-layer-deposition (ALD) technique. They then utilized these structures directly as an electrode for SC without any further modifications [138]. They additionally reported the application of molybdenum hexacarbonyl [Mo(CO)_6_], as a prototype emergent ancestor for low-temperature ALD fabrication, to deposit MoS_2_. To obtain molybdenum hexacarbonyl, a halide precursor (MoCl_5_) and H_2_S as a chemical reactant were deposited on the MoS_2_ films at an elevated temperature of 300 °C [138]. Therefore, Nandi et al. adopted hexacarbonyl precursor and H_2_S plasma as the chemical reactant. After a very comprehensive investigation of the properties of these nanomaterials and of their growth on the 3D Ni foam, the electrochemical studies were carried out, to realize the potential of plasma-enhanced atomic layer deposition (PEALD) MoS_2_ as an electrode in asymmetric SCs. The efficiency of the materials developed in terms of capacitance retention was reported, as shown in Figure 26. The newly developed method looks unique and simple for the synthesis of high-performance hybrid SC electrode materials.

It is difficult to review all published work related to MoS_2_ for SC applications, therefore we selectively summarize some excellent investigations on MoS_2_ based electrode materials, in addition to other graphene-based and hybrid composites, as enlisted in Table 8.

### 5.2. Polymer-Based Electrodes

Compared with metal/metal oxide nanocomposites, conducting polymers (CPs) show some advantages as electrode materials, such as low cost and ease of large-scale production [110]. Such hybrid nanocomposites for SC applications have been reviewed, and they generally showed high capacitive performance especially suitable for the growing demands for portable devices and hybrid electric vehicles. Certain CPs are particularly attractive because of their intrinsic conductivity and being conductive via a conjugated bond system along the polymer backbone. The most studied CPs are polyaniline (PANI), polypyrrole (PPy), and derivatives of polythiophene [88,150]. Based on these CPs, flexible SC device creation becomes possible. For example, Yang et al. reported flexible SCs fabricated using PANI-array-coated graphene electrodes, which offered a very impressive specific capacitance of 432.5 F/g at a current density of 1 A/g [151].

**Figure 27:**
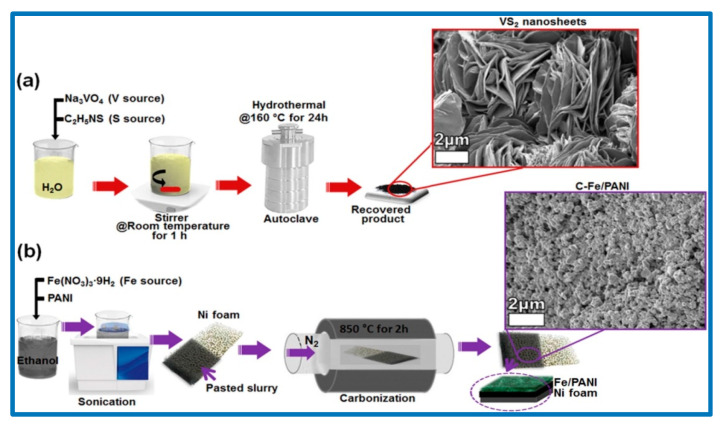
Schematic of the synthesis procedure for (**a**) VS_2_ nanosheets and (**b**) C-Fe/PANI as SC electrode materials (reproduced with permission from ref. [152]).

Additional hybrid CPs with metal/metal oxides as pseudocapacitive materials for SCs are also very promising. Rantho et al. successfully synthesized an asymmetric SC using VS_2_ nanosheets as a cathode and carbonized iron cations adsorbed on polyaniline (C-Fe/PANI) as an anode [152]. Using 6 M KOH as an electrolyte, they evaluated the electrochemical behavior of the working electrode in a three-electrode system. PANI is frequently used as the electroactive material in SCs owing mainly to its high conductivity and its variable oxidation states [153]. In their study, Rantho et al. selected Fe cations, which can be easily adsorbed onto PANI by complexation and electrostatic interaction, owing to the active binding sites presented in the functional groups on PANI surfaces [154]. They adopted a two-stage synthesis, first the creation of the VS_2_ nanosheets using a hydrothermal method, and then the fabrication of the C-Fe/PANI electrode material by direct pyrolysis of the Fe-PANI mixture coated onto a 3D Ni foam, in a tube furnace under N_2_ atmosphere. The synthesis procedure is depicted in Figure 27. Similar studies have also been carried by different research groups as discussed elsewhere [155,156].

**Figure 28:**
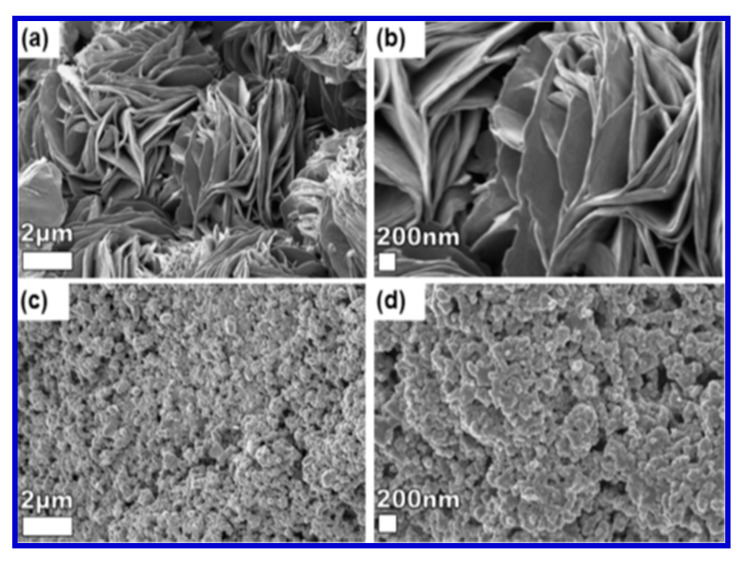
(**a**) Low- and (**b**) high-magnification FESEM micrographs of VS_2_ nanosheets. (**c**) Low- and (**d**) high-magnification FESEM images of C-Fe/PANI (reproduced with permission from ref. [152]).

The surface morphology of the nanostructured material was revealed by SEM analyses. Figure 28 shows the surface morphology of the samples obtained by Rantho et al., in which the VS_2_ sample was composed of a large number of nanosheets. However, in the case of C-Fe/PANI, as shown in Figure 28c,d, the crystallites were well distributed over the entire surface, indicating that the Fe cations were adsorbed uniformly over PANI.

The electrochemical characterization of the composite materials was carried out in 6 M KOH, and in Na_2_SO_4_. However, the results revealed that the composites had a better current response in 6 M KOH electrolyte, and the relevant explanation for such an observation was also suggested by Rantho et al. They reported the specific capacitance of 516.5 F/g for VS_2_ and 486.5 F/g for C-Fe/PANI. To further explore the electrochemical performance of VS_2_ and C-Fe/PANI electrodes, an asymmetric device was constructed by Rantho et al. The VS_2_/C-Fe/PANI asymmetric device offered a high-energy-density of 27.8 Wh/kg and a power density of 2991.5 W/kg at a current density of 2 A/g, as discussed in details elsewhere [152].

**Figure 29:**
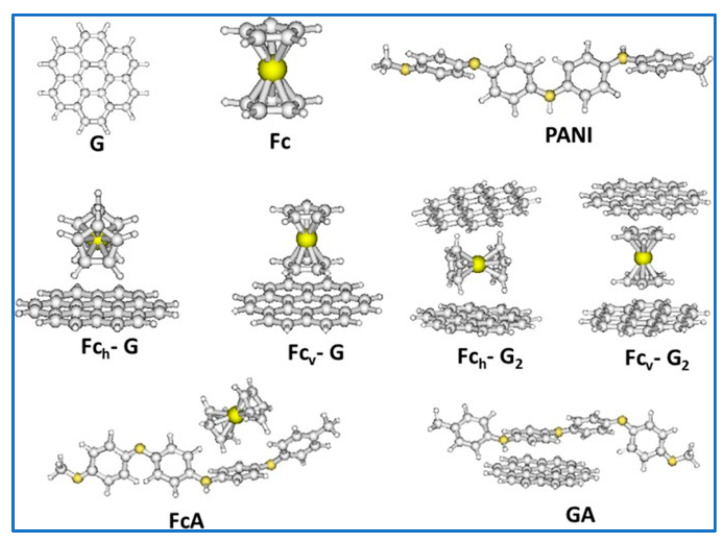
Relaxed structures for graphene (G), Fc, and PANI, and their possible interactions as modeled by DFT (reproduced with permission from ref. [106]).

In the same line, Adhikari et al. reported a polyaniline (PANI)-stabilized structure containing ferrocene (Fc) and graphene as the electrode material for SCs [106]. Their work highlighted the effective H bonding interaction between Fc and graphene and their stabilization by PANI, which occurs via the π–π interaction between PANI and graphene. DFT studies were also carried out that verified the successful interactions between the components. Table 9 along with Figure 29 summaries the DFT results of the synthesized composites [106]. For detailed bonding investigation, current experimental techniques have their limitations; therefore the attempt of DFT modeling could help to elucidate the interactions of various components and offer better guidance for materials selection and in-depth understanding for kinetic mechanism.

It is very important to note that supercapacitors using liquid electrolytes can provide better ion conductivity, but they also encounter several disadvantages, such as volatility, narrow potential window, and high flammability. Further, supercapacitors involving a liquid electrolyte do not offer the flexible feature and are generally bulky. For modern electronic gadgets, mobility and flexibility constitute the two essential characteristics. Hence, researchers are keen to seek suitable forms of electrolyte that offers high efficient, good flexibility, light in weight, and high mobility for SCs. The SC devices based on liquid electrolytes suffer from different disadvantages that limit their applications. These disadvantages include [154]:

- Leakage;
- Corrosion of electrodes;
- Limited geometry shape and high flammability.

Gel polymer electrolytes however can partly assuage the disadvantages of the liquid electrolytes, but the issues related to the liquid component in the electrolyte still exist, including the leakage and ionic reduction due to the solvent evaporation. The strategy that can be used to tackle these issues associated with the solvent based electrolytes is to develop solvent-free polymer solid electrolytes, which offer advantages, such as [157]:

- Improved stability, safety, flexibility and mechanical stability.
- Significantly streamline the packaging and make the specific separator needless.
- Improved gravimetric or volumetric energy densities of the devices.

## 6. All-Solid-State Supercapacitor

The increasing demands for wearable and lightweight energy-storage devices have brought about the necessity to design novel, efficient, and compatible energy-storage modes. Solid-state SCs (SSSCs) are proven to be very apt for applications in modern electronic gadgets. SSSCs are characterized by high power density and very stable cyclic performance with considerably fast charge/discharge rates [155,158,159]. The advantages of SSSCs can be summarized as follows:

(i) Facile electrode preparation: The electrodes can be easily fabricated by several approaches.

(ii) Easy electrolyte preparation that allows for the device compactness: Conventionally, liquid-electrolyte-based SCs are not practical options for mobile and compact electronic applications, as they are problematic to house the other elements and to achieve flexibility. In this context, the SSSCs are much more advantageous, since the all-solid-state property is not as itinerant as the liquid electrolytes, and the solid electrolyte can be easily kept separate from the other device parts.

(iii) SSSCs devices are perfectly compatible with modern-day flexible, lightweight, and compact electronic gadgets.

(iv) SSSCs can be fabricated into various flexible forms, such as thin films or fiber-shaped forms, or can be imbued with other novel properties. All these properties can widen the applications of SSSCs in many specific fields.

(v) Finally, and most importantly, SSSCs are equipped with properties like environmental friendliness, portability, mobility, and cost effectiveness, which can broaden the application area of SCs [155]. Therefore, SSSC devices are extremely necessary to expedite the growth of standalone microelectronic devices and to increase applicability in cutting-edge systems like various flexible and wearable electronic devices. However, to get the best performance, it is important to: (i) explore novel materials exhibiting high performance, (ii) improve the interfacial competence between the electrode and implemented electrolyte in the solid -state, and (iii) develop simple strategies for device fabrication. Hence, to improve the power- and energy-density performance of SSSCs, new advanced concepts need to be applied while designing SSSCs based on surveying novel materials with interesting architectures and properties [155,160]. The details about the advantages and overviews of graphene-based SSSCs over classic SCs have been discussed and reviewed recently by Liu et al. [161].

Recently, abundant carbon-based materials have been studied and evaluated for possible developments of SSSCs, in order to meet the demand of requisite energy storage. Example attempts include activated carbon [160,161], porous carbon [162], carbide-derived carbon [163], carbon nano-onions [164], carbon aerogels [165], carbon nanotubes (CNTs) [166], graphene [167,168,169], and graphene aerogel [170].

Among the above long list, graphene-based electrodes are much more attractive and have been widely investigated. Table 10 summarizes the different performance of various graphene-based SSSC electrode materials that were reported recently. High-quality mono- or multi-layered graphene sheets synthesized by methods such as chemical vapor deposition or mechanical exfoliation have shown high carrier mobility, but are proven unsuitable for SSSC applications, because the electrolytic ions cannot penetrate into the interlayers of graphene [155].

In addition, the yield of the aforementioned method is very low, and the cost involved in making graphene on a large scale remains high. A possible solution to the problem of large-scale synthesis of graphene is to use graphene oxide as a precursor. However, the performance of graphene oxide and reduced graphene oxide as electrode material is still limited, because of the limited surface areas of the electrode as a whole [184]. Zhang et al. have shown that the high-yield graphene obtained from molten salts using electrochemical exfoliation can also be used in the fabrication of flexible SSSCs [185]. However, the performance is still not good enough, because restacking issues of graphene needs to be tackled.

Various methods have been reported to assemble graphene into films, to prevent serious stacking aggregation, and to maintain their large specific surface areas while being lightweight. Khattak et al. reported the use of 3D (Fe_2_O_3_)/graphene aerogel hybrids as a flexible SSSC electrode, and achieved an impressive capacitance of 440 F/g [186]. Ghosh et al. fabricated a free-standing rGO-Co_3_O_4_ composite aerogel electrode, and reported a high areal energy density of 35.92 μWh cm^−2^ [187]. According to a report by Pedico et al., rGO aerogel decorated with Cu and Mo sulfides on carbon fibers acted as a high-performance wearable SC [188]. By adding oxide nanoparticles in the aerogel indeed can partly stop the restacking of graphene layers, since their existing as a spacer between layers hinders the direct contact of the neighboring layers. However, these processes are very tedious and need a lot of care during the electrode preparation and subsequent handling. Additionally, the raw materials used are quite expensive, which makes the devices less viable commercially. Finally, the interlayer charge transport remains a big challenge, too.

To eliminate the drawbacks of aerogels (like poor mechanical stability, tedious temptation process, expensive chemicals, supercritical drying, etc.) in SSSCs, several attempts have been documented to develop 3D hydrogels [189,190]. For example, Liu et al. recently demonstrated the fabrication of foldable all-solid-state SC using highly compressible 3D graphene hydrogel [189]. Other structural modifications, reported by He et al. [190], by integrating 1D Ni(OH)_2_ nanobelts with 2D graphene sheets, led to the formation of 3D composite hydrogel electrodes with high performance (1738.3 F/g at the scan rate of 10 mV s^−1^). Other attempts included the fabrication of 3D graphene aerogel first and then combining it with other redox materials, such as combining PANI and MnO_2_, to create all-solid-state flexible asymmetric SCs, which exhibited a specific capacitance of 111 F/g at the current density of 1 A/g [191].

Despite their various advantages, graphene-based electrode materials suffer from limited charge-storage capacity, owing to the surface storage mechanism, as seen in other carbon-based electrode materials. Moreover, the charge storage is largely limited by the unused electrode active surface of the graphene and by the large resistance to ion transport within graphene-based electrodes. Consequently, the value of the gravimetric capacitance of the reported SSSCs lies mostly below 300 F/g, far below its theoretical value [192]. The fabrication of the 3D graphene-based aerogel system is easy and cost-effective, but the performance needs to be improved [191].

**Figure 30:**
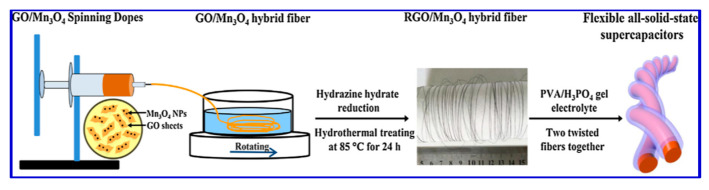
Schematic of the synthesis of rGO/Mn_3_O_4_ nanocomposite electrodes for SCs (reproduced with permission from ref. [194]).

To counter the above issues and boost the performance of graphene-based SSSCs, solutions including integrating graphene with other pseudocapacitive materials or doping it with various heteroatoms have been proposed [167,168,169,171,175,176]. In a recent report, Jadhav et al. demonstrated the design of MnO_2_ nanorods spread over the graphene surface, and reported a specific capacitance of 759 F/g [167]. Using fabric/polyaniline/graphene carbon woven composites, Lin et al. achieved an aerial capacitance of 790 F cm^−2^ at a current density of 1 A cm^−2^ [193]. Chen et al. used a facile approach to produce flexible electrodes for all-solid-state SCs, which exhibited an excellent volumetric capacitance [194]. They started with rGO/Mn_3_O_4_ nanocrystal hybrid electrodes and used a scalable wet spinning approach to achieve the flexible electrodes. By adopting this process, Chen et al. further revealed that the Mn_3_O_4_ nanocrystals were strongly anchored over the rGO nanosheets, which effectively avoided further aggregation of the nanocrystals. The regular fibrous-network-like structure was found to be favorable for flexible SCs [116]. The procedure for the preparation of Mn_3_O_4_ nanocrystal-based SCs is schematically presented in Figure 30.

**Figure 31:**
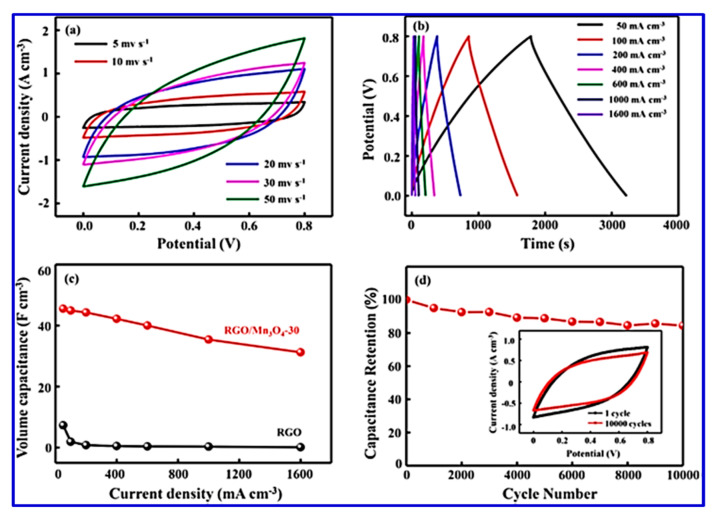
Electrochemical assessments of assembled all-solid-state rGO/Mn_3_O_4_-30 hybrid SCs. (**a**) CV curves at different scan rates from 5 to 50 mVs^−1^, (**b**) galvanostatic charge/discharge curves at different current densities from 50 to 1600 mA cm^−3^, (**c**) plots of volumetric specific capacitances at various current densities, and (**d**) cycle stability curve at a scan rate of 20 mVs^−1^ after 10,000 consecutive cycles (reproduced with permission from ref. [194]).

By varying the amounts of the Mn_3_O_4_ nanocrystals, the tensile strength, flexibility, and capacitive performance of the electrode materials were thoroughly investigated [194]. It was observed that the tensile strength of the electrode material decreased by increasing the amounts of Mn_3_O_4_ nanocrystals. The CV and EIS studies showed that the synergistic interaction between rGO sheets and the optimal amount of Mn_3_O_4_ crystals led to an excellent capacitive performance (Figure 31). The Mn_3_O_4_ crystals also offered an electrochemically active surface area with more electronic conduction channels. The flexible electrode offered a maximum volumetric capacitance of 311 F cm^−3^ in 1 M KOH electrolyte and 45.5 F cm^−3^ in PVA/H_3_PO_4_ gel electrolyte. It has good cycling stability of 85% over 10,000 cycles, and a high tendency to incur bending fatigue. Therefore, this approach can be considered as a successful example to achieve highly efficient energy-storage for the next generation of flexible electronics.

Numerous reports are available on SCs that use ionic, organic, and aqueous electrolytes. In the case of SSSCs, the electrolytes used are mostly in an aqueous or aqueous ion gel state, such as PVA/KOH, PVA/H_2_SO_4_, and PVA/H_3_PO_4_, and they are summarized above in Table 10. However, these aqueous gel electrolytes suffer from the problem of a narrow electrochemical window (0–1 V), which leads to a narrow cell voltage and therefore low energy and power densities [49,195,196]. These aqueous electrolytes also encounter the problem of water evaporation when operated at a wide temperature range, which affects the performance and the stability of the SCs. Compared with aqueous electrolytes, ionic liquids are advantageous as they are characterized with a wide electrochemical window (0–3.5 V), outstanding thermal stability, much-reduced volatility, non-flammability, and non-toxicity [49,197]. In particular, ionic-liquid-based electrolytes can indeed retain their liquid state even at room temperature. Further, the dissociated ions comprising them can also be used as an electrolyte and, hence, the device performance is not at all deteriorated. However, ionic electrolytes have their own shortcomings, such as reduced ionic conductivity. To date, the ionic conductivity of electrolytes has been understudied, and improving the ionic conductivity is, therefore, a very important goal. The ionic conductivity of electrolyte material depends significantly on surface chemistry and the specific surface areas of the material. rGO has been introduced into ionic electrolytes in an effort to improve the contact surface areas [49,198], and to enhance the ionic conductivity (reproduced with permission from ref. [199]).

**Figure 32:**
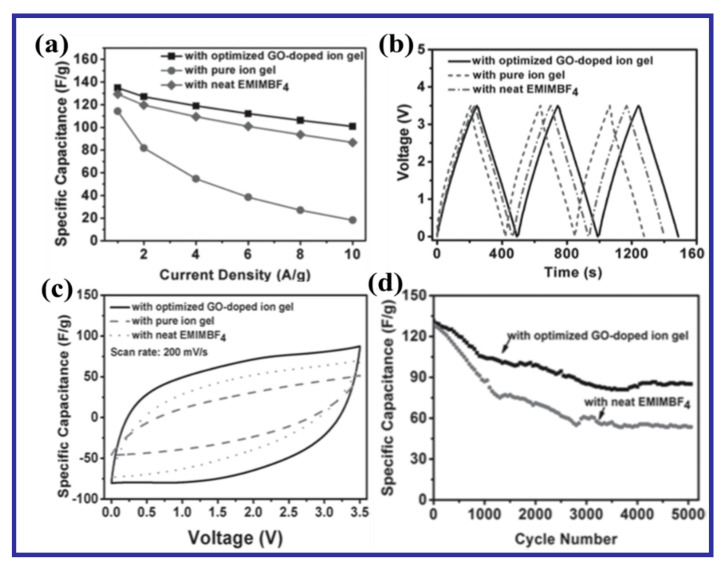
CV results of all-solid-state SCs with optimized GO-doped ion gel, pure ion gel, and conventional SCs with neat EMIMBF_4_. (**a**) Specific capacitances at various discharge current densities, (**b**) typical galvanostatic charge/discharge curves at a current density of 1 A/g, (**c**) comparison of CV analyses at a potential scan rate of 200 mVs^−1^, and (**d**) cycling performance at a charge/discharge current density of 1 A/g (reproduced with permission from ref. [199]).

**Figure 33:**
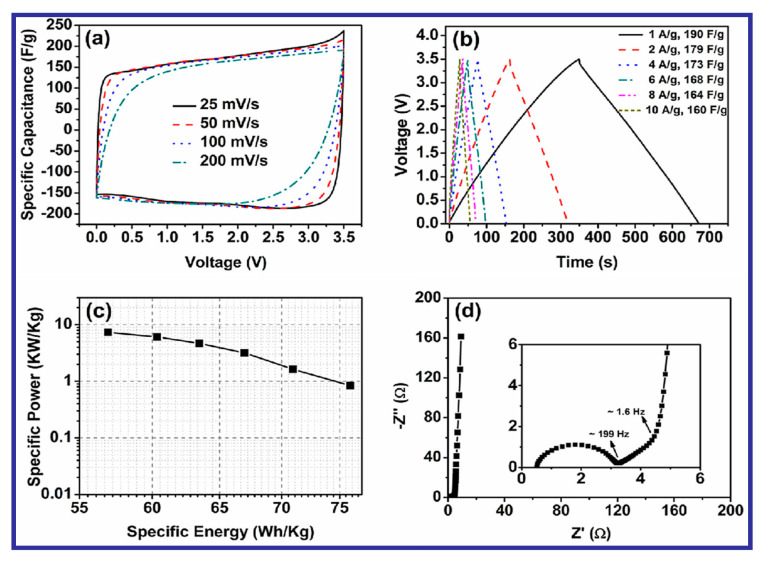
CV results of all-solid-date SCs. (**a**) CV curves obtained at different scan rates, (**b**) galvanostatic charge/discharge curves as obtained with application of different current densities, (**c**) Ragone plot obtained by plotting energy density and power densities, and (**d**) Nyquist plots showing impedance characteristics in the frequency range 10–100 kHz along with a magnified high-frequency region (reproduced with permission from ref. [201]).

**Figure 34:**
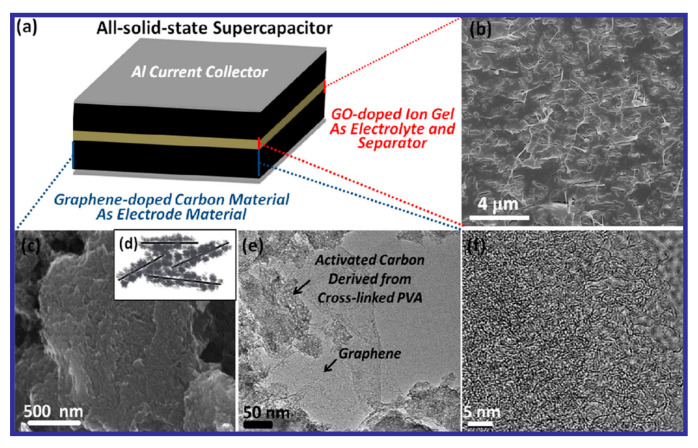
(**a**) Layers in the fabrication of all-solid-state SCs, (**b**) SEM morphology of GO-doped ion gel, (**c**) SEM image of graphene-doped carbon in which activated carbon is dispersed on graphene sheets, (**d**) acquired morphology of graphene-induced carbon, (**e**) TEM micrograph of graphene-doped carbon, and (**f**) HRTEM image of graphene sheets coated with activated carbon (reproduced with permission from ref. [201]).

Gao et al. reported a direct laser writing technique for the fabrication of rGO/GO micro-SCs in which GO was used as a solid electrolyte [200]. Yang et al. used GO-doped ion gel to improve ionic conductivity [199], and further showed that high-performance SSSCs can be fabricated using GO-doped ionic electrolytes acting as a gel polymer electrolyte and separator [200]. These reports have demonstrated that excellent performance of all-solid-state SCs could be achieved using GO-doped ion electrolytes to improve ionic conductivity and long-term stability (Figure 32). These SSSCs showed wide electrochemical windows (0–3.5 V; Figure 33) and significantly high ionic conductivity along with a specific capacitance of 190 F/g as discussed for the various systems elsewhere [200]. Figure 34 shows the various layers and materials involved in the fabrication of an all-solid-state SC and its performance [201].

**Figure 35:**
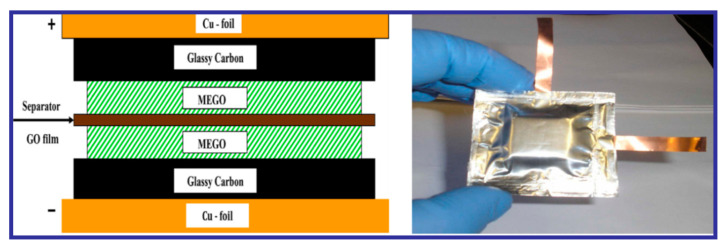
Scheme (**left**) for the fabrication of rGO-polymer hybrid SC (**right**; reproduced with permission from ref. [202]).

The use of rGO as a separator has been gaining research attention. Shulga et al. reported that they achieved a specific capacitance of 200 F/g by using such a separator [202]; their design is schematically shown in Figure 35. Ogata et al. also reported the fabrication of rGO/GO/rGO SSSCs with H_2_SO_4_-intercalated GO as electrolyte/separator by coupling with pseudocapacitive rGO electrodes [203]. This device exhibited excellent electrochemical performance with an aerial capacitance of 14.5 mF cm^−2^ and a volumetric energy density of 1.24 mWh cm^−3^.

## 7. Conclusions and Prospects

This review accounted for the recent progress in graphene and its 2D analogues based metal oxide/sulfide nanocomposites for applications in supercapacitors, with special focus on the developments incurred within the last 3–4 years. A comprehensive discussion has been included to link the surface morphology of the composites consisting of metal oxides, sulfides, metal foams, and metal containing polymers, with the performance for them acting as the electrode materials in SC applications. We have shown that these new nanocomposites have exhibited huge commercial applicability with easy processing features. A brief introduction to the basic principles for the supercapacitors has also been comprised, elaborated along with the current scenario in supercapacitors research. The properties of electrodes, separator, and electrolyte can be tuned separately and optimized together to realize ultimate improvements in the performance of the SCs. Furthermore, by using important schematic diagrams, figures, and tables, we created a comparative account on the state-of-the-art in SC research, so to help to achieve a deepened understanding and promote further thinking. By citing the most recent references in each section of this review, we have shown the ongoing cutting-edge research around the globe on supercapacitors, and also pointed out the potential directions where challenges remain for further exploration.

Graphene-based porous nanostructures are a class of materials of the utmost importance in the future development of high performance, portable, and flexible SCs. Since they can enhance the ion transportation rate in supercapacitors, consequently augmenting the power and energy density and the stability of supercapacitors. As recorded previously, rGO nanosheets working in aqueous H_2_SO_4_ or ionic BMIPF_6_ electrolytes have resulted in maximum capacitance values of 348 and 158 F/g, respectively, projecting the excellent performance of rGO in aqueous and/ionic electrolytes [22,27,28,29].

However, the cyclic stability remains a challenge, and it is prophesied to be improved along with the specific capacitance by the use of hybrid composites consisting of the newly discovered two-dimensional materials such as MXene and inorganic graphene [130,132,155,156]. The hetero elements doped graphene, GO, rGO, and graphene-based 3D porous nanostructures can all find use in the fabrication of next-generation electrodes [28,29]. Based on these structures, a high specific capacitance greater than 1500–2000 F/g has been recorded [155]. The high performance of rGO-filled liquid electrolytes is related to the oxygen-containing functional groups on the basal plane and edges of rGO, which affects the capacitance and lifetime performance of the electrolyte (18,000 cycles). Therefore, more research is required for their effective utilization in a commercial scale [155,159]. It is also possible to use GO paper as the separator, as reported that it achieved a very impressive specific capacitance of 200 F/g [155].

Nevertheless, it has been reported that graphene and other 2D material-based separators suffer from damages with time, which opens another big challenge for future study. Research in this field should also be more focused on their commercial viability and provide technical details towards their commercialization. Most importantly, flexibility based on nanostructures of graphene and its analogues, in the form of nanocomposites, paper sheets or aerogel, becomes possible, however commercial production poses a big task of the near future, mainly owing to the cost and technical barriers. Over-claim of their advantages and selectively ignore their shortcomings and realistic viability could be detrimental for building up consumer confidence and therefore for their wide commercialization.

Given the huge amount of joint efforts globally, the great investments in R&D, and the rapid paces of development in this area, we believe that these technical barriers and challenges could be solved in the short term. By combining the experimental selection of materials and parameters refinements with high-performance computer simulations at the molecular level, solutions to the challenges and optimizations to the performance of the SCs could be achieved much faster than working alone by materials scientists and chemists. We believe that SC, particularly low cost, lightweight, high power and energy density, flexible, and robust SCs will be realized very soon, not only in the lab but also produced in a commercial scale. We also believe that these next-generation SCs would contribute significantly to the future sustainable society.

## Figures and Tables

**Table 1:** Performance-related to various significant parameters for different energy-storage devices [2,10,12].

Characteristics	Batteries	SCs	Conventional Capacitor
Specific energy (Wh/kg)	10–100	1–10	<0.1
Specific power (W/kg)	<1000	500–10,000	>10,000
Discharge time	0.3–3 h	s to min	10^−6^ to 10^−3^ s
Charging time	1–5 h	s to min	10^−6^ to 10^−3^ s
Charge/discharge efficiency (%)	70–85	85–98	100
Cycle life	10,000	>500,000	Almost infinite

**Table 2:** Several frequently used aqueous electrolytes for SCs and their electrochemical performance.

Aqueous Electrolyte/Concentration	Electrode Materials	Specific Capacitance (F/g)	Cell Voltage	Energy Density (Wh/kg)	Power Density (W/kg)	Ref.
H_2_SO_4_/2 M	MMPGC	105 at 4 mV s^–1^	0.8	4	20	[55]
H_2_SO_4_/1 M	PANI-grafted rGO	1045.51 at 0.2 A/g	0.8	8.3	60,000	[56]
H_2_SO_4_/1 M	ANS-rGO	375 at 1.3 A/g	1	213	1328	[57]
KOH/6 M	p-CNTn/CGBs	202 at 0.325 A/g	0.9	4.9	150	[58]
Na_2_SO_4_/1 M	mesoporous MnO_2_	278.8 at 1 mV/s	1	28.4	70	[59]

**Table 3:** Several most frequently used organic electrolytes for SCs.

Electrolyte	Electrode Materials	Specific Capacitance (F/g)	Cell Voltage (V)	Energy Density (W h/kg)	Power Density (W/kg)	Ref.
1 M TEABF_4_/can	highly porous interconnected carbon nanosheets	120–150 at 1 mV s^–1^	2.7	25	25,000–27,000	[60]
1.6 M TEAODFB/PC	AC	21.4 at 1 A/g	2.5	28	1000	[61]
1.5 M SBPBF_4_/PC	AC	122 at 0.1 A/g	3.5	52	-	[62]
0.7 M TEABF_4_/ADN	AC	25 at 20 mV s^–1^	3.75	28	-	[63]
1 M TEABF_4_/PC	graphene–CNT composites	110 at 1 A/g	3	34.3	400	[64]
1 M LiTFSI/ACN	MnO_2_ nanorods–rGO/V_2_O_5_ NWs–rGO	36.9	2	15.4	436.5	[65]

**Table 4:** Different ionic liquids used in various supercapacitor applications.

Ionic Liquid	Electrode	Specific Capacitance	Cell Voltage	Energy Density	Power Density	Ref.
EMI-TFSI	TiC−CDC	160 F/g	3 V			[66]
BMI-PF_6_	CDC-950	125 F/g	1 V			[67]
[EMIM][Tf_2_N]	ACNT	440 F/g	4.28 V	148 Wh/kg	315 kW/kg	[68]
EMI-BF_4_	Graphene electrodes	250 F/g	4 V	53.1 Wh/Kg	9.838 kW/kg	[69]
BMI-PF_6_	Partially reduced graphene oxide	158 F/g	1.6 V	-	-	[70]
Et_3_NH TFSI	Diamond-coated Si nanowire	1.5 mF cm^−2^	4 V	-	25 mWcm^−2^	[71]
(PIP_13_-FSI)_0.5_(PYR_14_-FSI)_0.5_)	a-MEGO	150 F/g	3.5 V	-	-	[72]
BMI-TFSI was mixed with a Bi-redox IL (AQ-PFS^−^) (TEMPO^•^-MI^+^)	YP50	111 F/g	2.8 V	70 Wh/kg	-	[73]

**Table 5:** Comparative studies of selected most frequently studied electrode materials based on graphene nanocomposites and their electrochemical performance in SCs.

Electrode Materials	Capacitance (Rate, Electrolyte)	Energy Density	Power Density	Retention (Cycles)	Ref.
3D graphene-MnO_2_ composite networks	465 F/g (2 mVs^−1^, 0.5 M Na_2_ SO_4_)	6.8 Wh/kg	2.5 W/kg	81.2% (5000)	[92]
Mn_3_O_4_/RGO film	52.2 Fcm^−3^ (0.2 Acm^−3^)	18 mW h cm^−3^	3.13 W cm^−3^	100% (10,000)	[93]
PPy-NPG//MnO_2_–NPG	193 F/g (LiClO_4_)	86 Wh/kg	25 kW/kg	85% (2000)	[94]
NiO-GF//HPNCNTs	116 F/g (KOH)	32 Wh/kg	0.7 kW/kg	94% (2000)	[95]
NiCo_2_O_4_-rGO//AC	99.4 F/g (KOH)	23.3 Wh/kg	0.32 kW/kg	93% (2500)	[96]
Ni-Co LDHs	1766.4 F/g (1 A/g, 2 M KOH)	44.3 Wh/kg	0.425 kW/kg	85.05% (6000)	[97]
Graphene/NiAl–LDH	213.57 F/g (1 A/g, 1 M Na_2_SO_4_)	Not available	Not available	100% (1000)	[91]
Graphene fiber/3D graphene network	1.7 mFcm^−2^ (H_2_SO_4_-PVA)	1.7 × 10^−7^ Whcm^−2^	1 × 10^−4^ Wcm^−2^	Not confirmed	[98]
CNT-Graphene films	140 F/g (0.1A/g, 1 M H_2_SO_4_)		5.1 kW/kg	96.15% (2000)	[99]
Graphene fiber/3D graphene network	1.7 mFcm^−2^ (H_2_SO_4_-PVA)	1.7 × 10^−7^ Whcm^−2^	1 × 10^−4^ Wcm^−2^		[92]
PPy–NPG//MnO_2_–NPG	193 F/g (LiClO_4_)	86 Wh/kg	25 kW/kg	85% (2000)	[100]
NiO-GF//HPNCNTs	116 F/g (KOH)	32 Wh/kg	0.7 kW/kg	94% (2000)	[101]
NiCo_2_O_4_-rGO//AC	99.4 F/g (KOH)	23.3 Wh/kg	0.32 kW/kg	93% (2500)	[102]
3D ZnO/rGO/ZnO sandwich-structured	275 F/g (5 mVs^−1^, 1 M Na_2_SO_4_)	37.5 Wh/kg	26.9 kW/kg	98% (2000)	[103]
Graphene/NiAl–LDH	213.6 F/g (1 A/g, 1 M Na_2_SO_4_)	Not available	Not available	100% (1000)	[104]
Ni-Co LDHs	1766.4 F/g (1 A/g, 2 M KOH)	44.3 Wh/kg	0.425 kW/kg	85.05% (6000)	[105]
GNS/ PANI	1046 F/g (1 mV s^−1^, 6 M KOH)	39 W h/kg	70 kW/kg		[106]
CAN	331 F/g (5 A/g, 1 M KCl)	97.9 Wh/kg	1101.82 W/kg	92% (2,000)	[107]
CS@ZnO core–shell nanocomposite	630 F/g (2 A/g, 1 M)	Not available	Not available	70.80% (5000)	[108]
FcGA	960 F/g (1 A/g, 1 M NEt4BF4-acetonitrile)	85 Wh/kg	399 W/kg	86% (5000)	[109]

Typical abbreviations used in this table: NPG: nanoporous gold, HPNCNTs: N-doped hierarchical porous carbon@CNTs, GNS: graphene nanosheets, LDHs: layered double hydroxides, FcGA: PANI-stabilized ferrocene @graphene nanocomposite.

**Table 6:** Specific capacity and specific capacitance of BaMF_4_ systems.

System	Specific Capacity (mAh/g)	Specific Capacitance (F/g)	Equivalent Series Resistance (Ω)
BaCoF_4_	15.4	360	0.8
BaMnF_4_	30	200	1.4
BaNiF_4_	12.3	150	3.6

**Table 7:** A comparative summary of the specific capacitance of various nanocomposites based on LDH for SCs.

Electrode Materials	Electrode System	Type of Electrolyte	Working Electrode	Specific Capacitance	Ref.
MgAl LDH anchored rGO	three-electrode	1 mol L^−1^ KOH aqueous solution	graphite electrode	1334 at 1 A/g	[121]
Silver nanowire@hierarchical NiAL LDH	three-electrode	6.0 mol L^−1^ KOH	Ni foam (1 cm × 1 cm)	1246.8 at 1 A/g	[127]
NiAl LDH/CNT/Ni foam electrodes	three-electrode	1.0 mol L^−1^ KOH solution	Ni foam surface	1293 at 5 mA cm^−2^	[128]
Core–shell NiAl LDH	three -electrode	1 mol L^−1^ KOH aqueous solution	Ni foam substrate	735 at 2 A/g	[122]
NiAl LDH on Ni foam	three electrode	1 mol L^−1^ aqueous KOH solution	Ni foam (3 cm × 3 cm)	795 at 1 A/g	[123]
NiAl LDH	three -electrode	6.0 mol L^−1^ KOH solution	Ni foam surface	701 at 10 mA cm^−2^	[124]
Co3O4@NiCoAl LDH	three- electrode	6 mol L^−1^ aqueous KOH	Ni foam	1104 at 1A/g	[125]
CoAl LDH nanoflake	three -electrode	2 mol L^−1^ aqueous KOH	Ni mesh counter electrode	930 at 2A/g	[125]
CoAl LDH/rGO composite	three -electrode	6 mol L^−1^ aqueous KOH	Ni foam grids (1 cm × 1 cm × 0.2 cm)	479.2 at 1A/g	[126]
CoAl LDH nanosheets/rGO	three -electrode	2 mol L^−1^ aqueous KOH	Ni foam (1 cm × 1 cm)	1296 at 1A/g	[129]

**Table 8:** A comparative summary of recently developed high performance nanocomposite electrode materials containing MoS_2_, graphene, and other nanofillers.

SC Electrode Materials	Material Growth Process	Performance		Ref.
		Areal Capacitance	Cycling Stability	
MoS_2_@CNT/rGO	hydrothermal	129 mF cm^−2^ at 0.1 mAcm^−2^	94.7% after 10,000 cycles	[139]
NiS@Ni-foam	electrochemical deposition	2640 mF cm^−2^ at 2.35 A/g	95.7% after 2000 cycles	[140]
PANI/carbon cloth	electro-polymerization	3300 mF cm^−2^ at 1.1 A/g		[141]
FeCo_2_O_4_@Ni-foam	chemical deposition	1880 mF cm^−2^ at 2 mA cm^−2^	91% after 5000 cycles	[142]
MoS_2_/CoS_2_ nanotube arrays on Ti plate	hydrothermal	142.5 mF cm^−2^ at 1 mA cm^−2^	92.7% after 1000 cycles	[143]
Monolayer 1H-MoS_2_@oleylamine	hot-injection thermolysis	50.65 mF cm^−2^ at 0.37 A/g	240% after 5000 cycles	[144]
Ni*_x_*Co_3−_*_x_*S_4_ nanosheet@NiCo_2_O_4_ nanowire arrays grown on carbon fiber paper	hydrothermal followed by electrodeposition	1860 mF cm^−2^ at 1 mA cm^−2^	87.6% after 10,000 cycles	[145]
NiCo_2_O_4_@Ni-foam	solvothermal followed by calcination	925 mF cm^−2^ at 5 mA cm^−2^	75.8% after 6000 cycles	[146]
NiO/MnO_2_@carbon cloth	hydrothermal followed by chemical deposition	286 mF^−2^ at 0.5 mA^−2^	89% after 2200 cycles	[111]
MoS_2_-graphene composite	ultrasonication	11 mF cm^−2^ at 5 mV s^−1^ scan rate	∼250% after 10,000 cycles	[147]
MoS_2_ thin films	chemical vapor deposition	71 mF cm^−2^ at 1 mV s^−1^ scan rate		[148]
rGO/Ni_0.3_Co_2.7_O_4_	hydrothermal followed by calcinations	2370 mF cm^−2^ at 2 A/g	>100% throughout 7000 cycles	[149]
MoS_2_@3D-Ni-foam	ALD	3400 mF cm^−2^ at 3 mAcm^−2^	82% after 4500 cycles	[138]

**Table 9:** Sample designations and interaction energy.

System	Interaction Energy (kcal mol^−1^)
Ferrocene@graphene with ferrocene kept horizontal to graphene (Fc_h_-G)	−1.78
Ferrocene@graphene with ferrocene kept vertical to graphene (Fc_v_-G)	−0.45
H–π interactions for sandwiched geometries with ferrocene placed between two graphitic sheets (Fc_h_-G_2_)	−2.74
π–π interactions for sandwiched geometries with ferrocene placed between two graphitic sheets (Fc_v_-G_2_)	−0.93
ferrocene@polyaniline (FcA)	1.72
graphene@polyaniline (GA)	−17.08

**Table 10:** Various solid states SCs (SSSCs) based on graphene and their performances reported recently.

Electrode Materials	Electrolyte	Specific Capacitance	Cell Voltage (V)	Energy Density	Power Density	Ref.
MnO_2_/graphene/carbon fiber	Na_2_SO_4_	759 F/g	1	42.7 Wh/kg	22.5 kW/kg	[167]
V_2_O_3_/N-rGO	LiCl/PVA	216 mF cm^−2^	0.6	0.55 mW h cm^−3^	0.035 W h cm^−3^	[168]
GNF/PNT	3-M KCl	128 mF cm^−2^	0.8	11.4 μWh cm^−2^	720 μW cm^−2^	[169]
GH/PANI	1-M H_2_SO_4_	311.3 F/g	1	66.3 Wh/kg	539.9 W/kg	[171]
rGO/MnO_2_ nanosheets	PVA-LiClO_4_	46 mFcm^−2^	1.6	48.8 mWh cm^−3^	8.34 W cm^−3^	[172]
THAQ/rGO	H_2_SO_4_ gel	259 F/g	1	17 μWh cm^−2^	164.0 μW cm^−2^	[173]
nitrogen-enriched active carbon fiber/rGO	PVA/KOH gel	283 F/g	1	35.2Wh/kg	399.1 W/kg	[161]
MoO_3_/GF	PVA/KOH gel	136 F/g	1.6	51.91 Wh/kg	0.838 kW/kg	[174]
rGO/Ag NW@NiAl-LDH	PVA/KOH gel	127.2 F/g	1	35.75 mWh cm^−3^	1.01 W cm^−3^	[175]
WO_3_/G/PT	PVA-H_2_SO_4_ gel	308.2 mF cm^−2^	0.8	60 μWh cm^−3^	2.32 mW cm^−3^	[176]
MoS_2_/rGO	6M KOH	472 F/g	0.8	-	-	[177]
(rGO/CNTs) @PANI	PVA/H_2_SO_4_ gel	193.4 F cm^−3^	0.8	0.98 mWh cm^−3^	16.25 mW cm^−3^	[178]
rGO/MWCNT	PVA/H_3_PO_4_ gel	46.6 F cm^−3^	2	6.47 mW h cm^−3^	10 mW cm^−3^	[166]
CNY@PPy@rGO	PVA/H_3_PO_4_ gel	80.46 F cm^−3^	1	-	-	[179]
mPPy@rGO-POM	PVA/H_2_SO_4_ gel	115 mF cm^−2^	1	4.8 mW h cm^−3^	645.1mW cm^−3^	[180]
rGO/PANI	PVA/H_2_SO_4_ gel	0.92 F cm^−2^	1	-	-	[181]
CNT/CF/rGO/MnO_2_	1-M Na_2_SO_4_	356 F/g	1	-	-	[182]
PANi/rGA/AgNPs	6-M NaOH	365.14 F/g	0.9	64–116 Wh/kg	1550–7944 W/kg	[183]

Typical abbreviations used in this table: THAQ: 1, 4, 5, 8 tetrahydroxy anthraquinone, CNY: carbon nanofibers yarns, GF: graphene foam, NW: nanowire.

## Abbreviations

SCs	Supercapacitors
ED	Energy density
PD	Powder density
EDLC	Electrical double-layer capacitor
ESR	Electrochemical series resistance
SSSCs	Solid-state SCs
CNT	Carbon nanotubes
GO	Graphene oxide
RGO/rGO	Reduced graphene oxide
HCNS	Carbon hollow nanospheres
LDH	Layered double hydroxide
CNFs	Carbon nanofibers
CPs	Conducting polymers
PANI	Polyaniline
CV	Cyclic voltammetry
DT	Discharge time
CT	Charging time
GQD	Graphene quantum dot
ACN	Acetonitrile
PC	Polycarbonate
1D, 2D, and 3D	Dimension
DFT	Density-functional theory
GNF	Graphene nanoflake
GNS	Graphene nanosheets
PANI	Polyaniline
